# Computer-assisted instruction versus inquiry-based learning: The importance of working memory capacity

**DOI:** 10.1371/journal.pone.0259664

**Published:** 2021-11-09

**Authors:** Johann Chevalère, Loreleï Cazenave, Mickaël Berthon, Ruben Martinez, Vincent Mazenod, Marie-Claude Borion, Delphine Pailler, Nicolas Rocher, Rémi Cadet, Catherine Lenne, Norbert Maïonchi-Pino, Pascal Huguet

**Affiliations:** 1 Laboratoire de Psychologie Sociale et Cognitive (LAPSCO), Université Clermont Auvergne et CNRS, Clermont-Ferrand, France; 2 Laboratoire d’Informatique, de Modélisation et d’Optimisation des Systèmes (LIMOS), Université Clermont Auvergne et CNRS, Clermont-Ferrand, France; 3 Rectorat de Clermont-Ferrand, Clermont-Ferrand, France; 4 Laboratoire Activité, Connaissance, Transmission, Éducation (ACTé), Université Clermont Auvergne, Clermont-Ferrand, France; 5 Maison pour la Science/Auvergne (MPSA), Université Clermont Auvergne, Clermont-Ferrand, France; 6 Laboratoire Physique et Physiologie Intégratives de l’Arbre en Environnement Fluctuant (PIAF), Université Clermont Auvergne et INRAe, Clermont-Ferrand, France; National Taiwan University of Science and Technology, TAIWAN

## Abstract

The Covid-19 pandemic has led millions of students worldwide to intensify their use of digital education. This massive change is not reflected by the scant scientific research on the effectiveness of methods relying on digital learning compared to other innovative and more popular methods involving face-to-face interactions. Here, we tested the effectiveness of computer-assisted instruction (CAI) in Science and Technology compared to inquiry-based learning (IBL), another modern method which, however, requires students to interact with each other in the classroom. Our research also considered socio-cognitive factors–working memory (WM), socioeconomic status (SES), and academic self-concept (ASC)–known to predict academic performance but usually ignored in research on IBL and CAI. Five hundred and nine middle-school students, a fairly high sample size compared with relevant studies, received either IBL or CAI for a period varying from four to ten weeks prior to the Covid-19 events. After controlling for students’ prior knowledge and socio-cognitive factors, multilevel modelling showed that CAI was more effective than IBL. Although CAI-related benefits were stable across students’ SES and ASC, they were particularly pronounced for those with higher WM capacity. While indicating the need to adapt CAI for students with poorer WM, these findings further justify the use of CAI both in normal times (without excluding other methods) and during pandemic episodes.

## Introduction

The accelerating spread of Covid-19 has led the majority of countries across the globe to close their schools for varying lengths of time. If closing schools seems to be a logical decision in order to impose social distancing in the population, it tends to have a disproportionately negative impact on academic learning over time, with half of students affected worldwide [1]. This reality has left a poor impression on digital learning (countries used television, radio, online platforms, and take-home packages [2]) as inequalities have increased as a result of the digital divide [3, 4], but more importantly, due to the lack of an education expert where many responsibilities and tasks were imposed on parents [5]. In order to prevent future scenarios, it is important to focus on methods that guarantee autonomous learning trough interaction with digital expert tutors able to monitor the learning process efficiently to mitigate learning loss. Off-pandemic times should therefore be exploited to examine the efficacy of such methods compared to other innovative and more popular methods involving face-to-face interactions. The present study sought to investigate this question in Science and Technology, by examining the benefits of computer-assisted instruction (CAI) compared to a different, although well-established and well-defined instructional method for teaching science topics, which does not traditionally rely on digital technologies, that is, inquiry-based learning (IBL). We were also interested in examining the possible moderating role that socio-cognitive factors, and especially working memory (WM), may play in this respect.

### Literature review

#### Computer-assisted instruction

Computer-assisted instruction refers to a self-learning method using computers where instruction adopts training techniques monitored to meet specific needs and tailored to a student’s pace [6]. CAI has long been a subject of research, starting with a large body of studies which emerged after the computer revolution in the sixties [7, 8]. Surprisingly however, CAI is still rarely used on a large scale today [9]. This tendency is, however, expected to change as the recent worldwide pandemic episodes have stressed the need to encourage these forms of instruction in order to prevent the disastrous consequences of school closure [10]. It is thus timely and relevant to provide an updated view of the effectiveness of such methods in order to prepare for future scenarios.

Thanks to half a century of progress since the pioneering work of Carbonell [7], CAI now permits the deployment of a wide-ranging content, both informative and evaluative through a variety of high-quality virtual materials (texts, videos, graphics, audios) relating to specific topics [11]. The training methods used in CAI may include drill-and-practice exercises that reinforce basic skills through repeated exposure to content [12], problem-solving exercises [13], tutorials that make use of hints and feedback [14], simulations that translate conceptual content into realistic situations [15], educational games where the learner playfully competes with the computer [16] and interactive stories that propose narrative scenarios where student and the computer form a partnership to complete the task [17].

*The effectiveness of computer-assisted instruction*. Research into the effectiveness of CAI can be broken down into two distinct time periods, corresponding to the first and second generations of CAI, which were endowed with different degrees of adaptivity and artificial intelligence. As documented in a meta-analysis of 50 experimental studies [8], both old and more recent research has usually compared computer tutoring to individualized human tutors or to teacher-led instructions, referred to as no-tutor conditions. The first generation of computer tutors produced moderate positive effects of about .30 standard deviations relative to no-tutor conditions [18]. The second generation, more sophisticated and adaptive and referred to as intelligent tutoring systems, showed greater effectiveness from .37 to .66 [8, 19, 20].

To evaluate the effectiveness of CAI, the choice of comparison group is crucial and the CAI intervention is usually contrasted with a group receiving so-called “conventional classroom instruction” [8], or “teacher-led large group instruction” [18]. However, the notion of “teacher-led large group instruction” is a coarse definition of what goes on in the classroom and the transmission of knowledge may vary greatly depending on the teachers’ characteristics or their preferred methods of instruction. A better comparison should include instruction based on more identifiable and well-defined practices for the domains under interest here, that is, Science and Technology.

#### Inquiry-based learning

A particularly well-suited method for teaching scientific subjects is IBL. It refers to a self-learning method that follows practices similar to those of professional scientists in order to construct knowledge through self-directed investigations [21]. The famous philosopher John Dewey and the Physics Nobel Prize-winner Georges Charpak democratized IBL by promoting the application of scientific reasoning principles to education in order to provide students with the skills required in modern societies. Typically, the method consists in teachers supervising a group of students working collaboratively in face-to-face interactions and elaborating scientific concepts using hypothetico-deductive thinking [22, 23]. When addressing a particular science topic, the IBL process is based on the active reproduction–by students themselves–of the fundamental steps of scientific reasoning: formulating hypotheses, designing an experiment, collecting results, interpreting them and drawing conclusions [24, 25]. To do so, students work hands-on in dedicated classrooms, thus allowing them to interact with physical material, under the guidance of a trained teacher who ensures that the IBL reasoning phases are completed successfully.

**The effectiveness of inquiry-based learning** has long been a subject of debate. While the evidence from the early studies indicated positive effects of IBL compared to more conventional, teacher-led instruction [26, 27], international reports have challenged this position. The 2015 PISA [28, 29] report investigated achievements in science among students from primary to middle school across all countries and economies included in the project. Regarding the impact on IBL on academic performance, the PISA report showed that after statistically controlling for students’ and schools’ socioeconomic status (SES), IBL was negatively associated with students’ performance in fifty-six countries. When the analysis was restricted to the OECD countries, IBL was positively associated with features other than performance, such as epistemological convictions and motivation to engage in a scientific career, even though these correlations were weaker than with direct instructional methods. Providing a fine analysis of PISA 2015 in England, Jerrim et al. [30] reached similar conclusions. The analysis showed that, unless associated with high levels of guidance, IBL had a very weak positive relationship with attainment in science, and that this small effect was robust regardless of the type of inquiry, test measure, varying levels of disciplinary climates in classrooms, gender, and prior attainment.

Indeed, guidance is an important variable that seems to account almost entirely for the presence of an IBL effect [25, 31]. Applying a meta-analytic approach to 72 studies, Lazonder and Harmsen [25] distinguished among studies using different types of guidance and contrasted these studies with others using unguided IBL. They found that a minimal amount of guidance was needed for IBL to be effective, with an average effect size of .50 on learning outcomes in terms of learning skills and domain knowledge. The fact that the IBL size effect on learning skills was twice as high (.78) as that on domain knowledge (.37) is consistent with previous findings suggesting that IBL is particularly suited for improving epistemological thinking rather than memorizing factual content [29], and better suited for deep than surface learning [32]. Furthermore, increasing the degree of guidance (i.e., from mere supervision to full explanations) seemed to have little impact except on measures of performance during ongoing activities [25], suggesting that any type of guidance is sufficient to elicit an IBL effect on learning outcomes. However, it should be remembered that Jerrim et al. [30] found that IBL was effective only when coupled with high levels of guidance. Finally, Lazonder and Harmsen [25] showed that the positive effect of guided IBL was not specific to age, suggesting that children, teenagers and adolescents benefited equally from it.

#### Comparing the two methods

**Fig 1:**
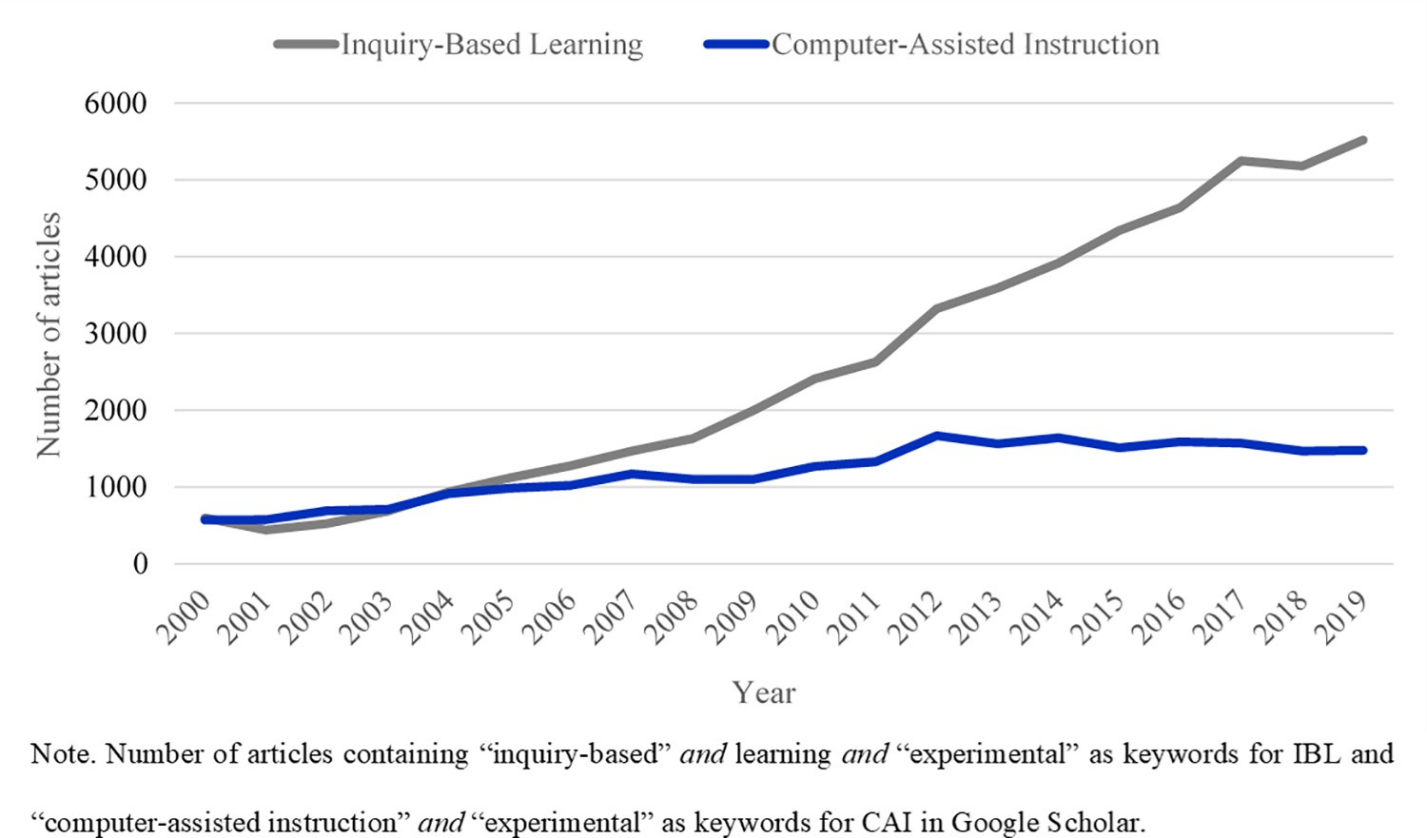
The popularity of IBL and CAI over the past twenty years.

Although the popularity of IBL has risen more steeply than that of CAI in recent years (cf. Fig 1), this does not necessarily mean that IBL is more effective. Curiously, the two methods have never been compared directly in a single integrative study on identical objects of knowledge in the Science and Technology field. Our approach is oriented towards helping teachers to identify which methods, among available ones, are most efficient in inculcating knowledge and competences that reflect standard evaluation criteria from the national programme. It is worth noting that in the general curriculum, those criteria largely involve the acquisition of factual and conceptual knowledge more than meta-cognitive skills [33]. Therefore, in the absence new evaluation policies, a method’s efficiency is here understood as its ability to address those standard criteria. In the context of the digital revolution, and given the considerable financial supports available for digital technologies in education (EdTechs) [34], it is critically important to determine whether CAI is beneficial compared to well-established and well-defined alternative forms of instruction such as IBL. The CAI vs IBL comparison is interesting as both methods a) are typically associated with active learning. In both methods, students play an active role in the learning process by engaging in problem-solving activities, an approach which requires more than just listening [35, 36]. Furthermore, in both methods b) students benefit from highly interactive environments and c) work autonomously in a self-paced manner under the supervision of the teacher [17, 37].

Conversely, CAI and IBL differ with regard to three main factors. These concern a) the use of digital technology for learning (absent in IBL) and b) the collaborative nature of IBL practices. In IBL, students indeed typically engage in peer to peer interactions [23], while in CAI, they typically interact with the computer agent [38]. Therefore, in IBL they can engage in debates and discussions, activities which are minimized in CAI. Another difference concerns c) the immersive properties and frequent use of private feedback in CAI which are known to provide high control over the task and keep students busy and motivated [39]. Feedback in CAI is delivered by the computer thus not visible by the peers. In contrast most feedback in IBL is public, especially those coming from peers. It should be noted that IBL has also been adapted for use in computer-assisted environments [40, 41]. In the present study, however, we are interested in the very pragmatic and direct comparison of CAI vs IBL in their differing but conventional forms as applied to identical objects of knowledge. This comparison was prompted by the following simple and pragmatic question: which of the two methods, in their conventional form, is more effective for teaching identical topics in Science and Technology?

#### The role of working memory, academic self-concept and socioeconomic status

Not only has no direct comparison ever been made, but there is also a striking lack of documentation on how basic socio-cognitive individual differences–fundamental in education–may modulate the effectiveness of the two methods. The intrinsic navigational nature of CAI and its rich functionalities and content raise questions regarding the cognitive requirements that can support such a form of instruction, especially in terms of WM capacity [42, 43]. WM is thought of as a flexible but limited mental capacity that permits the temporal maintenance and manipulation of information in an active state for ongoing processing [44]. It reflects “an ability to maintain information in the maelstrom of divergent thought” [45], where *maintenance* relates to the crucial ability to temporarily store information in memory while directing attention towards the stimuli that are relevant to our current goals (e.g., learning). WM is a strong predictor of general cognitive abilities [45, 46] and academic achievement [47, 48]. More precisely, WM is essential for supporting complex activities such as language, reading comprehension, problem-solving and reasoning [49, 50].

Students with low WM capacity (i.e., hence, those who are less able to control their attention) might be particularly impacted by the diversity of content, materials and navigational features of CAI, which may distract them from their learning goals. The digital environments implemented in CAI indeed expose students to large amounts of information presented in different modalities and through hypertext links that may overload the cognitive system [51, 52], especially in students with low WM capacity who may need to repeat the same action several times in order to understand specific pieces of information before moving on to the next step. As an illustration of the deleterious effects of navigational features, Scharinger et al. (2015) [53] found an additional cognitive load when reading involved having to navigate through hypertext links compared to pure reading.

For different reasons, IBL could be equally challenging for the attentional system. According to Cognitive Load theorists (e.g., [42, 54]), classic IBL relies little on previous explicit exposure to content, thus preventing novice students from building a mental model of the material itself in long-term memory [55, 56]. This may result in IBL instructional designs that increase cognitive load [55, 57] and impair retention [58]. However, providing high levels of guidance in IBL reduces cognitive load [55] and the more complex the task is, the more guidance is required [57].

Additionally, the present study considered two other major socio-cognitive factors that may moderate the CAI/IBL effects, namely academic self-concept (ASC) and SES. Academic self-concept, the perception that students have about their own abilities compared with those of their classmates [59], constitutes one of the most relevant variables in the academic world because of its influence on motivation, learning and cognitive functioning [60]. As IBL requires students to work collectively, those with a low ASC may experience negative social comparisons with some of their classmates and lack the necessary confidence when reasoning in their presence [61]. This in turn may hamper their progression due to increased confusion or increased social withdrawal under IBL, a problem that may be reduced under CAI. This idea is supported by evidence showing that publicly drawing attention to the failures of students with low ASC—even without any intention to force a negative comparison with their peers- may cause new failures to arise [61]. An alternative hypothesis holds that collaborative work may be a means to improve self-efficacy [62], a construct close to ASC [63]. In particular, students who deliberately pay attention to peers who succeed in the task at hand are likely to increase their sense of self-efficacy [62, 64].

Students’ socioeconomic background may also make a difference. Those from privileged backgrounds may have access to more opportunities to explore sciences outside the school (e.g., family support, going to museums, having encyclopaedias and personal computers at home) [65], thus enhancing their knowledge and potentially giving them an advantage in both methods compared with their low-socioeconomic counterparts.

### Research questions

The aim of the present study was to compare the two methods on identical Science and Technology topics taken from the official French national educational programme, while also focusing on socio-cognitive factors, WM in particular, as possible moderators of the effects of these instructional methods. More precisely, we sought to answer the following questions:

1. Is CAI more effective than IBL in learning similar topics in Science and Technology?
2. Do WM capacity, ASC and SES modulate the effects of these instructional methods?

## Materials and methods

### Participants

An initial sample of 837 middle-school students participated in this study. Of the initial sample of students, 4.2% did not complete the academic tests in at least one of the disciplines, including Physics-Chemistry, Earth and Life Sciences, and Technology. Of the remaining 802 participants, 26.6% made errors on more than 50% of the secondary task of the WM task (see the “Working memory capacity” subsection of the Materials) and were therefore excluded from the analyses. We additionally excluded 5.8% of the remaining 589 students as they were identified as univariate outliers on WM performance on at least one of the two following criteria: interquartile range*1.5 and Cook’s distance. Of the remaining 555 participants, 8.3% did not complete all the items of all the ASC scales in the related disciplines. The final sample therefore consisted of 509 middle-school students (*M*_*age*_ = 12.82, *SD* = 0.44; 272 females), which is quite large compared to experimental field studies in these areas [8, 25]. All of the students were seventh graders, 282 took all three courses (55%), 97 took two courses (19%), and the remaining 130 took only one course (26%). Within the final sample size (N = 509), 46% were categorized as privileged students and 54% were disadvantaged students according to the nomenclature of professions and socio-professional categories of the French Ministry of Education. Three-hundred-and-twenty-eight students (64%) received IBL instruction and the remaining one-hundred-and-eighty-one students (36%) were taught using CAI. Since our research project took place in authentic school settings, the level of participation of particular schools and referent teachers determined the enrolment of individuals or groups of students in one or more topics.

### Ethics statement

The study is part of a larger research project which received an approval from the Clermont Auvergne University Ethics Committee (number IRBO0011540-2018-08) in conformity with the French law on bioethics (covering Psychology). All participants’ parents received a written informed consent form several weeks before the study that they had to read and sign to allow their child to participate.

### Lesson plan and implementation

#### Computer-assisted instruction implementation

The CAI versions used for each subject and topic (“mass and volume” in Physics-Chemistry, “climate” in Earth and Life Sciences, and “material structure” in Technology) came from recent versions of a tool (Tactileo©) developed by Maskott©. These more sophisticated and dynamic versions were the product of a collaborative project in which programmers integrated the material content provided by teachers while parametrizing the CAI in accordance with teachers’ and researchers’ recommendations. For this study, we adopted the idea that technologies created or adapted by research teams and including teachers are more efficient for learning than those either taken from the commercial market or that simply use the technology as a delivery system [66]. For each topic, secondary education teachers, school inspectors, programmers and designers collaboratively transcribed the knowledge content of specific topics taken from the official French national educational programme into the system. The selection of the knowledge content was decided on and supervised by state school inspectors representative of the three disciplines involved. The knowledge content was adapted to the CAI architecture by means of a variety of pedagogical training methods that are typically reported in the CAI literature [13, 14, 17], including problem solving exercises and tutoring modes embedded in narrative scenarios (see S1 and S2 Appendices in S1 File). The content was displayed through a variety of materials (texts, videos, audios). The teachers’ role was to introduce the topic and then to let their students learn on their own by interacting with the CAI and only intervene in the case of problems or questions from students. Students were instructed to avoid collaboration with other students in order to maximize the time spent at their computer. However, if students spontaneously interacted with their classmates, the teachers did not stop them from doing so as long as the exchange was brief. The teachers therefore encouraged their students to interact primarily with the computer. Each student assigned to the CAI condition was equipped with a digital tablet and interacted with the CAI in their usual classrooms in the presence of their usual subject teacher.

#### Inquiry-based learning implementation

All teachers in the IBL condition had been trained in this method by experts from a national foundation dedicated to IBL, represented locally by the “House for Science in Auvergne” (Maison Pour la Science en Auvergne). This training was a prerequisite for teachers to be involved in the IBL condition and guaranteed that they met the national standards for good IBL practices, including being able to give an appropriate level of guidance to students (c.f. [30]). The underlying content knowledge in the IBL condition was identical to that of the CAI condition and adapted to the instructional method by expert IBL teachers under the supervision of state school inspectors. In the same way as in the CAI condition, teachers in the IBL condition introduced the topic and provided general guidelines on how to reason scientifically about the topics under study. Students worked in their usual classrooms and were instructed by their teachers to learn on their own by collaborating with other students (in groups of 3–4 students) according to the IBL guidelines of the House for Science in Auvergne. Teachers intervened in the case of problems or questions from students, while also ensuring that the different phases of IBL reasoning (i.e., formulating hypotheses, designing an experiment, collecting results, interpreting them and drawing conclusions) proceeded correctly.

#### Intervention duration

**Fig 2:**
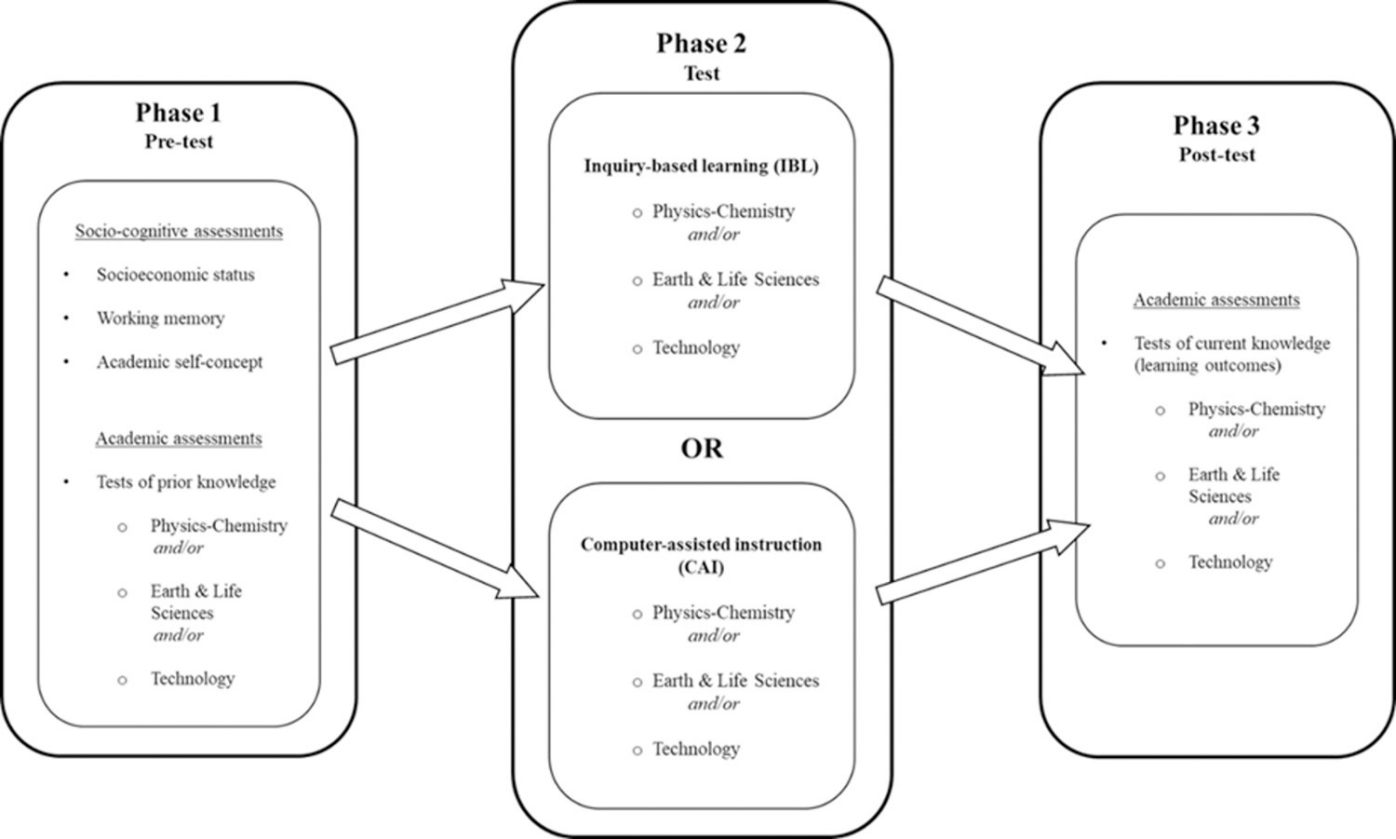
The experimental design of the present study. The pedagogical material and academic test metrics in Physics-Chemistry, Earth and Life Sciences, and Technology were collaboratively elaborated by teams of teachers from each discipline. All data were collected in real classroom environments via an online platform dedicated to the present research, including demographics, academic tests, questionnaires, and WM measures.

In both the IBL and CAI conditions, students were exposed to topics related to Physics-Chemistry, Earth and Life Sciences, and Technology (see Fig 2) for a period varying from four to ten weeks. More precisely, the exposure duration to CAI and IBL differed across disciplines depending on the usual amount of time devoted to each topic in the French national educational programme. The CAI and IBL interventions lasted four weeks for Technology (focusing on material structure), six weeks for Earth and Life Sciences (climate), and ten weeks for Physics-Chemistry (mass and volume).

### Materials

#### Academic performance

*Test of prior knowledge (T0) before intervention*. Measures of prior knowledge were taken in order to determine the efficacy of CAI on academic performance in Physics-Chemistry, Earth and Life Science and Technology. These measures assessed contents from the national educational programme acquired during the previous year and were used to provide a performance baseline intended to control for initial individual differences in performance as well as to examine potential interactions with instructional methods. The tests of prior knowledge consisted in short-answer questions and multiple-choice questions focusing on relevant topics from the national educational programme for each subject. Students took the T0 at least two weeks before the experimental interventions. Both to maximize statistical power and to standardize test metrics, the three T0 scores were centred, averaged and scaled to form composite Science and Technology scores ranging from 0 to 20 points.

*Tests of knowledge (T1) after intervention*. As for T0, T1 measures also consisted in short-answer and multiple-choice questions focusing on relevant topics of the national educational programme for each subject (for an example of a T1 knowledge test, see S3 Appendix in S1 File). In contrast with T0, T1 measures assessed contents that were taught during the current curriculum year (seventh grade) via participation in one of the two instructional methods (IBL or CAI). The T1 tests included a mixture of factual knowledge and learning skills in accordance with the requirements of the French government (see S4 Appendix in S1 File), which was represented by state school inspectors who actively collaborated in this study. In Physics, for example, the students had to learn to differentiate the notions of mass and volume, to understand which instruments are used to measure one or the other, and their conditions of use. The “paths” for this learning were therefore different depending on whether the students were exposed to the CAI or IBL method. However, the final test was composed of questions and exercises corresponding to the common denominator of the knowledge and skills that each student could, in principle, acquire with these two methods. Students took T1 approximately two weeks after the intervention. Again, the three T1 scores were merged into a single Science and Technology score ranging from 0 to 20 points.

#### Socio-cognitive assessments

*Working memory capacity*. This continuous variable was the WM performance score on the Operation span task adapted from [67] and available online at http://englelab.gatech.edu/taskdownloads. The Operation span task is a computer-based task consisting of lists of to-be-remembered (TBR) items interspersed with to-be-processed (TBP) items. Participants had to memorize lists of TBR items while processing the items in the secondary task and to recall the lists of TBR items at the end of each trial. The TBR items were letters and the secondary task was an arithmetic operation judgment task. For each TBP item, participants had to click on “yes” or “no” response buttons to determine whether the current item was correct or incorrect among an equal number of correct and incorrect items. At the end of a trial, a response screen invited participants to recall the TBR items in serial order by clicking on the right items presented among a number of distracters and then press an “enter” button to validate the response. The WM score corresponded to the average proportion of memory items (i.e., consonants) that were correctly recalled in serial order for lists of 4, 5 and 6 consonants.

*Academic self-concept*. A 6-point Likert-type scale [68] was adapted from the French translation by Huguet et al. (2009) [59]. We modified Huguet et al.’s version developed for French and Mathematics to assess self-concept in Physics-Chemistry, Earth and Life Sciences, and Technology. All three versions showed very good reliability (Cronbach’s alphas > .80). Academic self-concept scales were tailored to each discipline; therefore, if students were taking more than one course, their ASC in related disciplines were averaged.

*Socioeconomic status*. We used the nomenclature of professions and socio-professional categories published by the French National Institute for Statistical and Economic Studies [69]. We collapsed the original four-category indicator (i.e., disadvantaged, medium, privileged to highly privileged backgrounds) into two categories (i.e., low and high) in order to simplify the statistical analyses.

### Data collection

All these data (academic performance and socio-cognitive assessments) were collected online via a dedicated platform built for the purpose of the study. Students completed the tests and questionnaires directly on the platform, which was made accessible from the school computer lab. Data collection was supervised by national education personnel trained for the purpose of the study. During data collection, each class was divided into two groups to guarantee a sufficient number of computers per student and minimize noise.

Data collection spanned a maximum of thirty-seven weeks and varied depending on each Science and Technology discipline. At the beginning of the school year, all students completed the psychological assessments over a period of four weeks. Several weeks later (two to four weeks for Technology, nine to eleven weeks for Earth and Life Sciences and thirteen to-fifteen weeks for Physics-Chemistry), the academic pre-tests were administered. Two weeks later, the intervention was deployed for four to ten weeks (see Intervention duration). Finally, two weeks after each intervention, the academic post-test was administered.

### Data analyses

We applied multilevel random intercept models to the three-level structure of the data (509 students in 48 classes in 11 schools). Random attribution to experimental conditions (i.e., IBL, CAI) was simply not feasible here since an optimum CAI approach depended on essential equipment-related conditions, such as an adequate internet connection or a sufficient number of modern computers per student, that not all schools could fulfil. This is a typical field constraint found in many large-scale studies [70]. To overcome this constraint, we followed up-to-date recommendations [71, 72] and performed multilevel modelling, while carefully controlling a range of parameters in order to increase validity by reducing estimation bias, as described below. We conducted all the statistical analyses with R software version 4.0.1 [73] and used the CAR [74] and lme4 [75] packages for the preliminary analyses and subsequent multilevel models, respectively.

## Results

### Preliminary analyses

**Fig 3:**
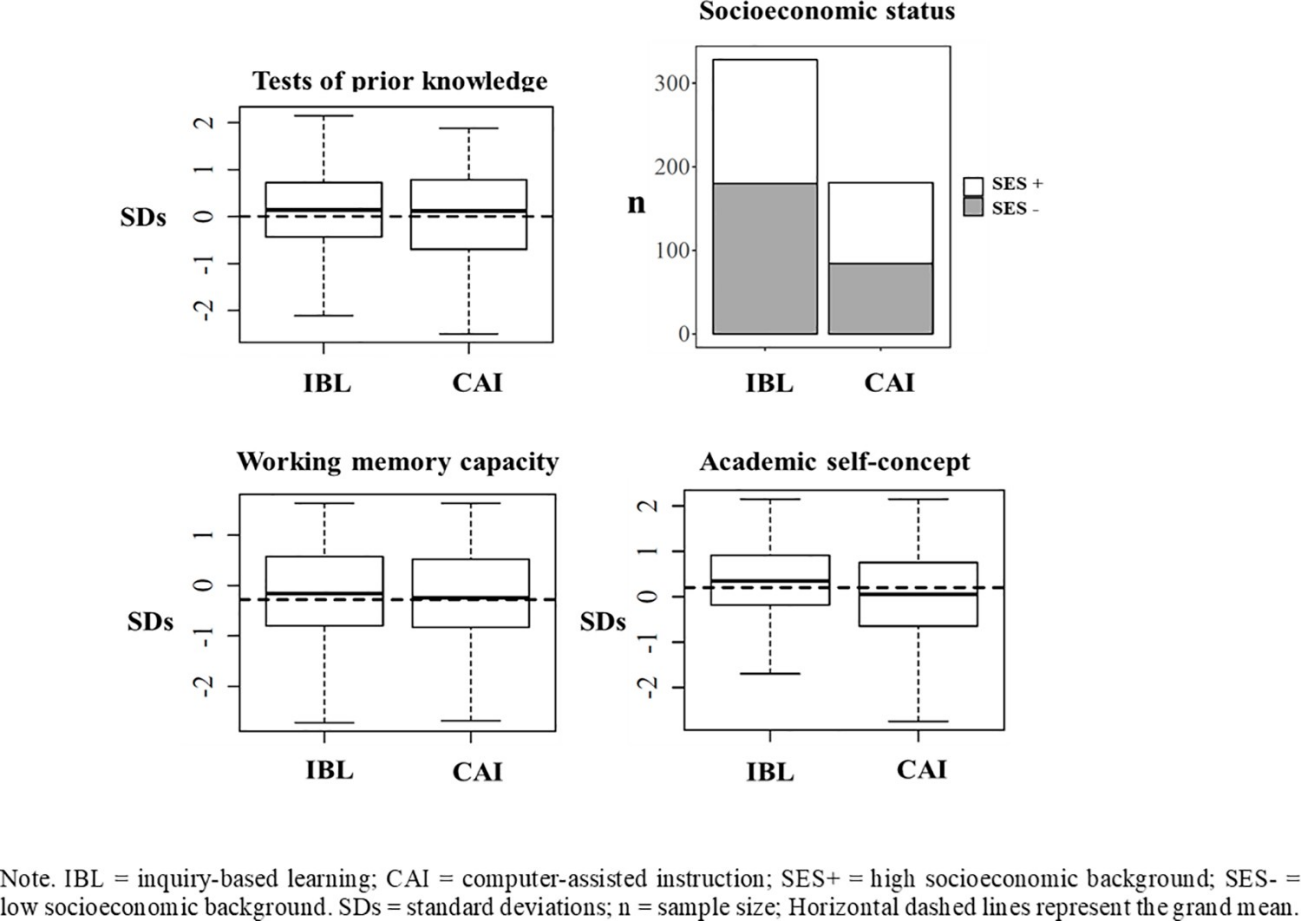
Pre-test imbalance analysis for measures of prior knowledge, socioeconomic status, working memory capacity and academic self-concept.

We conducted preliminary analyses of pre-test imbalance (Fig 3) for students’ prior knowledge, SES, WM and ASC to ensure that students exposed to IBL and CAI had similar socio-cognitive characteristics. These analyses resulted in non-significant group differences for prior knowledge, *F*(1, 507) = 1.31, *p* = .25, SES, χ^2^ (1) = 3.02, *p* = .08, and WM, *F* < 1. Only ASC showed an imbalance at pre-test, *F*(1, 507) = 17.09, *p* < .001; however, as the effect size was very small (η^2^ = .03), it was easily dealt with in the subsequent statistical procedure by fixing all covariates at their grand mean [71, 72]. Prior knowledge and socio-cognitive factors were entered as covariates and fixed at their grand mean in subsequent multilevel analyses. This procedure allowed us to obtain bias-free estimates of the effects of the instructional methods on learning outcomes.

### Multilevel models

**Fig 4:**
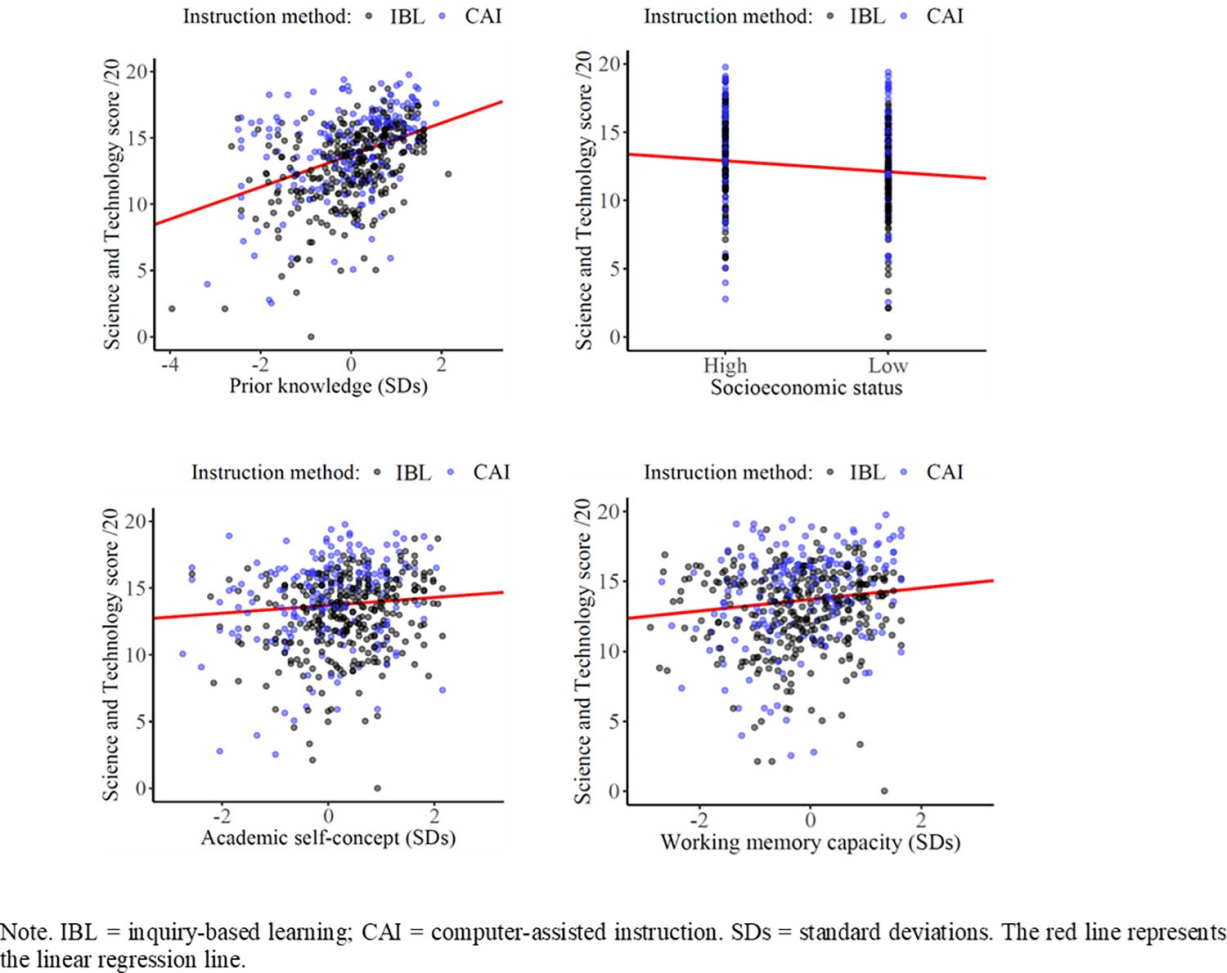
Estimated regression coefficient effects of prior knowledge, socioeconomic status, working memory and academic self-concept on Science and Technology scores (Model 4).

Out of a series of models testing either main effects of instructional methods, prior knowledge and socio-cognitive factors, or moderation effects in addition to main effects, two models fitted the data equally well (see Table 1). Of the two models (Models 1 and 4), Model 1 comprised main effects of instructional method, prior achievement, and socio-cognitive variables, whereas Model 4 additionally comprised a moderation effect of instructional method due to WM capacity. As both models showed equal utility, we focused on the more explanative, interaction model (Model 4). The percentages of variance (Intra-Class Correlation coefficients, ICCs) explained by schools and classes out to the total variance were negligibly small (ICC_schools_ = 0.5% and ICC_classes_ = 0%) and are therefore not depicted. Importantly, as shown in Fig 4, prior knowledge and each socio-cognitive variable independently contributed to learning outcomes.

**Table 1:** Random intercept multilevel models in Sciences and Technology for the IBL vs. CAI comparison.

n students = 509, n classes = 48, n schools = 11	Model 0		Model 1		Model 2		Model 3		Model 4		Model 5	
	*E*	95% CI	*E*	95% CI	*E*	95% CI	*E*	95% CI	*E*	95% CI	*E*	95% CI
**Fixed Effects**												
*Student Level*												
Intercept	13.22	[12.75;13.75]	13.68***	[13.35;.13.99]	13.66***	[13.29;14.01]	13.69***	[13.36;14.00]	**13.71** ***	**[13.40;14.04]**	13.68***	[13.31;14.00]
T0			1.22***	[.93;1.48]	1.16***	[.90;1.45]	1.22***	[.94;1.49]	**1.21** ***	**[.92;1.44]**	1.22***	[.93;1.50]
SES			-.78**	[-1.24;-0.29]	-.76**	[-1.31;-.30]	-.71*	[-1.28;-.05]	**-.80** **	**[-1.31;-.35]**	-.78**	[-1.26;-.24]
WM			.22	[-.04;.49]	.23	[-.04;.47]	.23	[-.04;.48]	**.41** **	**[.13;.70]**	.23	[.001;.48]
ASC			.31*	[.04;.61]	.31*	[.05;.60]	.31*	[.04;.58]	**.30** *	**[.04;.57]**	.31**	[.01;.60]
*School Level*												
CAI			1.29**	[.68;1.86]	1.28**	[.65;1.87]	1.30**	[.79;1.83]	**1.38** **	**[.79;1.97]**	1.29**	[.64;1.89]
*Cross Level Interactions*												
CAI x T0					-.23	[-.75;.30]						
CAI x SES							.22	[-.82;1.35]				
CAI x WM									**.57** *	**[.06;.1.12]**		
CAI x ASC											-.01	[-.61;.51]
**Random Effects**												
*Residual variance*												
Student Level (*SD*)	9.65	(3.11)	7.48	(2.73)	7.46	(2.73)	7.48	(2.74)	**7.42**	**(2.72)**	7.45	(2.74)
Class Level (*SD*)	.00	(.00)	.00	(.00)	.00	(.00)	.00	(.00)	**.00**	**(.00)**	.00	(.00)
School Level (*SD*)	.47	(.69)	.04	(.19)	.04	(.22)	.04	(.19)	**.04**	**(.19)**	.04	(.19)
Log Likelihood	-1305.0			-1235.0		-1235.2		-1235.5		**-1233.3**		-1235.6
AIC	2618.1			2489.1		2490.3		2491.0		**2486.6**		2491.1
BIC	2635.0			2527.2		2532.6		2533.3		**2528.9**		2533.5
Χ^2^ (*df*)	138.95 (5)			**-**		.83 (1)		.18 (1)		**4.56 (1)**		.002 (1)
*P*	< .001			**-**		= .36		= .67		**= .03**		= .96

Note. The model in bold is the preferred model. T0 = test of prior knowledge; SES = socioeconomic status; WM = working memory; ASC = academic self-concept; CAI = computer-assisted instruction (effect); E = estimate; CI = confidence interval; SD = standard deviation; df = degrees of freedom; AIC = Akaike information criteria; BIC = Bayesian information criteria

* = significant at *p* < .05

** = at *p* < .01

*** = at *p* < .001. Model 1 (main effects only) was the reference for comparison with all other models. Model comparison was based on a combination of fit indicators, namely, X^2^*p*, AIC and BIC values. The model with the lowest AIC and BIC values along with a significant X^2^*p* indicated best fit for the data and was therefore preferred. The AIC value indicated that Model 4 best fitted the data while the BIC value (penalizing for model complexity) indicated a better fit for Model 1.

Values of the regression coefficients from Model 4 for main effects of prior knowledge, SES, WM and ASC are shown in Table 1, Model 4. Fig 4 shows adjusted regression lines. Each effect was estimated at the grand mean of the other factors. The following factors positively predicted academic performance: prior knowledge, β = 1.21, *p* < .001, 95% CI [.92; 1.44], WM, β = .41, *p* = .008, 95% CI [.13; .70], and ASC, β = .30, *p* = .04, 95% CI [.04; .57]. Socioeconomic status negatively predicted academic performance, β = -.80, *p* = .001, 95% CI [-1.31; -.35]. As described below, only WM capacity modulated the effects of instructional methods.

### Is computer-assisted instruction more effective than inquiry-based learning in learning similar topics in Science and Technology?

**Fig 5:**
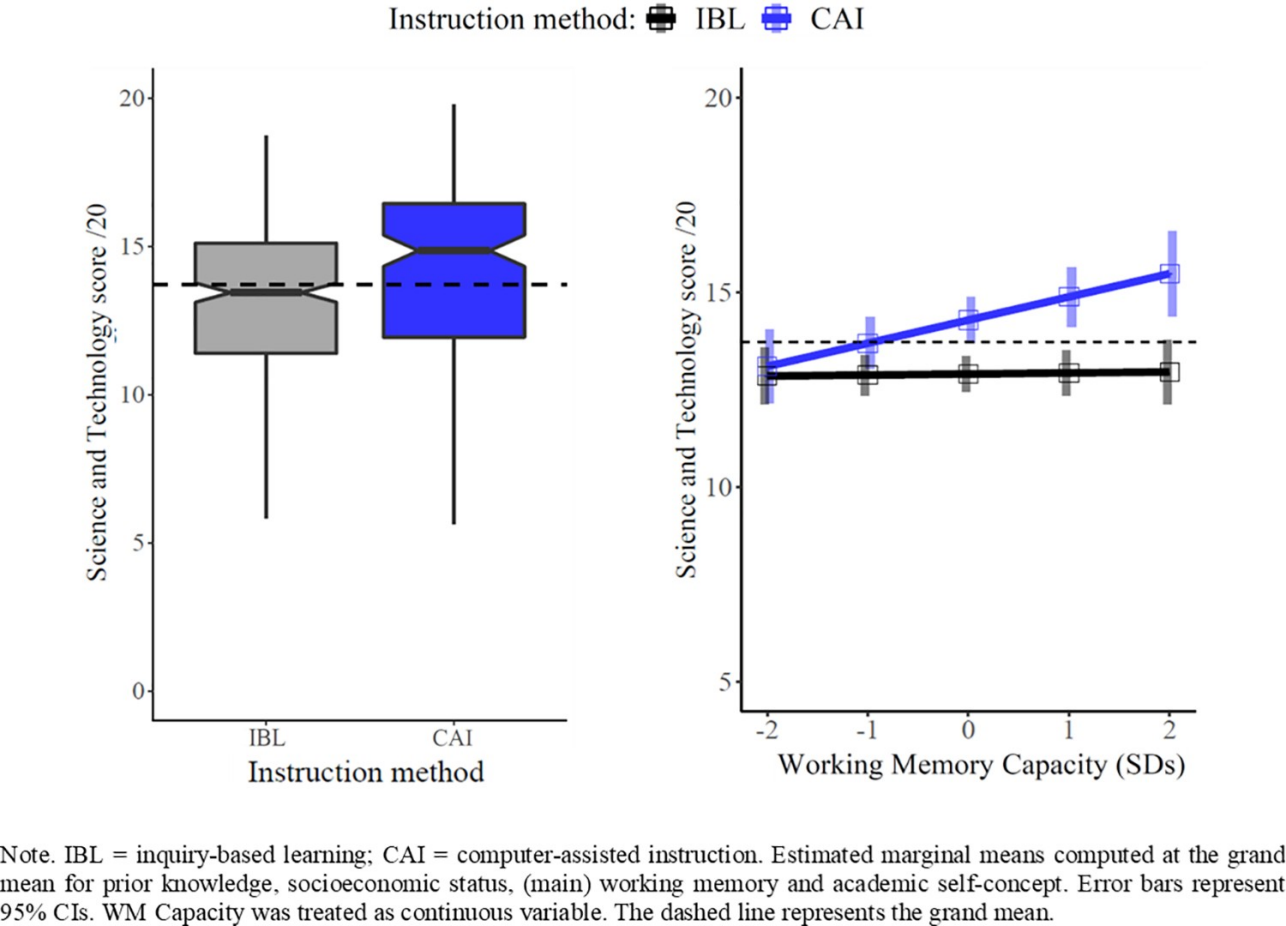
Adjusted mean Science and Technology scores (Model 4) in inquiry-based learning and computer-assisted instruction (left panel) and as a function of students’ working memory capacity (right panel).

Fig 5 (left panel) displays the significant main effect of instructional methods (β = 1.38, *p* = .001, 95% CI [.79; 1.97]) found in Model 4, Table 1. The results revealed that students who received CAI significantly outperformed students who received IBL in Science and Technology by 1.38 points (out to 20). This roughly corresponds to an improvement from the 50^th^ to the 68^th^ percentile. In other words, average students without any special commendation would be eligible for a *cum laude* distinction if instructed with CAI as opposed to IBL. Regarding the prevention of school failure, these results indicate that about 6% of students receiving IBL who failed on national evaluations would have succeeded with CAI. Six percent of seventh graders still represents a population of nearly 50,000 students in France [76] and 300,000 students in the US [77].

### Do working memory capacity, academic self-concept and socioeconomic status modulate the effects of instructional methods?

Fig 5 (right panel) shows the significant Instruction Method * WM capacity interaction effect (β = .57, *p* = .03, 95% CI [.06; 1.12]) found in Model 4, Table 1. A one-standard deviation gain in WM capacity resulted in a supplementary CAI benefit of 0.57 points (out to 20) regardless of students’ prior knowledge, SES, and ASC. This additional benefit corresponds to an approximate improvement from the 68^th^ to the 78^th^ percentile. This means that among students who failed on national evaluations, an additional 2% would have succeeded if taught with CAI instead of IBL, thanks to their higher WM capacity of about one standard deviation above average. Transposed to the general population of seventh graders, this proportion would represent 15,000 and 90,000 students in France [76] and in the US [77], respectively.

## Discussion

The PISA 2015 [29] survey (i.e. a survey conducted every 3 years with a sample of more than 500,000 middle-school students in 72 participating countries) reported no link between financial investments in information and communication technologies for education and students’ results on standardized tests, a finding which has revived the debate about the effectiveness of digital devices in education [10]. However, the PISA (2015) report is based on non-experimental and cross-sectional data. While the correlational analyses reported in the PISA surveys may question the usefulness of digital practices, they are not sufficient to invalidate the relevance of such practices in education. Furthermore, digital education represents an umbrella term for very different methods where sophisticated tutoring approaches such as CAI are lumped together with less sophisticated ones that merely deliver content, which limitations in terms of effectiveness have been put forward by the Covid-19 events [5].

### Computer-assisted instruction was more effective than inquiry-based learning on students’ performances in Science and Technology

In the present comparative experimental approach, our findings suggest that CAI generally outperforms IBL in Science and Technology, with the benefits being greater for students with higher WM capacity. Although it is beyond the scope of this article to determine all the characteristics responsible for the better performance observed in CAI, there may be several explanations. First, the highly-structured nature of CAI, supported by a variety of training methods, helps keep students engaged in the learning process and avoid off-task behaviours [78]. Conversely, research has shown that collaborative work (such as in IBL) is prone to off-task behaviours [79] and may lead to great variability in within-group individual contributions to the task [80] both having negative consequences on learning outcomes [81]. Second, and more importantly, the tutoring modes in CAI ensure structured support adapted to each student’s learning pace [82]. This entails more regular feedback than in IBL simply because teachers working with large classes have limited time and attention, making it difficult to support each student individually [83]. Although student peers may provide some degree of feedback during IBL, the quality of this feedback may not be as valid and reliable as a teacher’s expert feedback [84]. Third, the narrative scenarios in CAI may produce more contextualized representations in long-term memory and foster meaning attribution [85, 86] although this element is less likely to make a difference as the IBL environment is highly contextualized too and additionally provides physical interaction with the real word, which is known to enhance memory retention [87, 88].

The finding that CAI outperforms IBL is an important one since IBL is considered a gold standard method for teaching Science and Technology at school. In these domains, in which reasoning, planning and finding solutions through face-to-face collaborative interactions–all of which are central to IBL–are the rule rather than the exception, our results are counter-intuitive. Our findings do not mean that these highly desirable practices are not efficient. Instead, they mean that allowing students to reason and plan alone with CAI may also be a valuable option at certain points in students’ learning trajectories, meaning that teachers should be able to include CAI in their repertoires along with other alternatives. An optimally balanced combination between different instruction methods may follow a dynamic adjustment to match individual needs and temporary states during the learning process. For example, performing CAI prior to IBL might help a student gain more confidence before being confronted with others’ opinions while the opposite might give another student the opportunity to reflect on previous actions and discussions when receiving feedback from the digital tutor agent.

Another lesson learned from our data is that CAI proved superior regardless of student’s ASC and SES, indicating that many students may indeed derive the same benefits from it. This lesson is more encouraging than the correlational reanalyses reported in the PISA 2015 data [89] which suggest that increasing the use of digital technologies for educational purposes among students who are less likely to use these technologies benefits only students of medium and high SES. By focusing specifically on the effectiveness of CAI by means of an experimental approach, we challenge this position by suggesting that CAI may help bring about greater equality of learning opportunities among students from different socioeconomic backgrounds. Likewise, the fact that CAI was of equal benefit to students with high and low ASC echoes what has been reported experimentally in undergraduate students receiving either face-to-face or online instructions [90].

### The benefit of computer-assisted instruction was higher for students with a higher working memory capacity

Interestingly, we found a greater benefit of CAI in students with higher WM capacity. Given that the content was identical across the CAI and IBL conditions, we interpret the observed effect as being a consequence of the instructional design. Students’ WM might be overloaded by the complexity of the CAI environment, with the result that students who are better able to overcome this difficulty benefit more. In particular, the navigational nature of CAI, including hypertext links and tools that enhance student’s autonomy, might distract attention from essential information that will not be properly assimilated by the attentional system [43]. The navigational demands of CAI particularly affect the extraneous component of cognitive load, that is, the complexity of task-irrelevant material associated with the way information is presented (cf. the instructional design), as opposed to intrinsic load, that is to say the complexity of the information itself [42, 91].

Another possibility is that IBL could have made greater demands on WM by increasing the cognitive load [55]. However, this account is not supported by the significant Instruction Method * WM capacity interaction effect, which indicates that CAI imposes greater WM demands. There are two possible explanations for this. First, the IBL teachers in our study were trained to meet the national standards for IBL. These include an appropriate level of guidance [25, 30], which reduces the cognitive load [55, 57] and therefore also the WM demands of the task. The second explanation is based on the collaborative nature of IBL and transactive memory. Transactive memory refers to a collective mechanism through which a group develops a memory system that distributes information across partners [92]. In collaborative tasks, the interaction between partners seeking to achieve a common goal often results in a specialized division of labour where the different partners adopt specific roles in the task [92, 93]. In a first encoding phase, the partners’ roles are defined [94], for example, different members perform the different scientific steps involved in IBL. During a storage phase, the members store the information specific to their roles, thus retaining as opposed to sharing different information [92, 95]. During a retrieval phase, the members combine the different sources of information that have been encoded and stored within the framework of their respective roles. Consequently, each partner works as a memory aid for the others, leading to a collaborative memory system that exceeds the capacity of each individual member [92, 93]. As retention is distributed across partners, the cognitive load for each individual, and thus the WM demands of the IBL instructional design for each individual learner, may be reduced. Given that students work individually in CAI, WM demands are higher since each student must memorize the content on their own, making the contribution of WM more visible.

To help reduce school failure through AI -during both normal and troubled times- our findings suggest that one important aspect requiring attention is the consequences of CAI use for students with below-average WM capacity, for whom CAI brought no benefit (see Fig 2, right panel, students with -2 standard deviations from the mean WM measure). This suggests that the conditions of use of CAI should be adapted for these students, who are more likely to exhibit attentional problems. In line with previous recommendations, one solution may be to shorten or sequence the CAI session (e.g., 15-min sessions, 3 times per day) in order to relieve attention and memory load [96]. Fortunately, this objective could easily be achieved since CAI is highly flexible, individualized and remains accessible outside of school. Furthermore, adaptations could be made based on the instructional design of CAI according to each student’s WM capacity. To reduce the cognitive load for all students and further boost the benefits of CAI, our CAI condition should have carefully considered the split attention effect [42, 97]. When synchronized in a way that maximizes multimodality overlap, the use of different media modalities helps focus students’ attention, for example through the complementary and simultaneous inclusion of audio and visual sources [42, 97]. For students with lower WM capacity, decreasing the number of elements presented on screen in the light of individual capacity may reduce the observed WM effect [93, 98]. However, considering that students with higher WM capacity can process larger amounts of information, thus deriving more benefit from CAI, we may still expect the achievement gap between low and high WM students to increase even with more WM-adaptive CAI technology. This can be viewed as an extension to CAI of the Matthew effect, a framework describing how children with various minor advantages in reading (and other abilities) progress faster and draw away from their less advantaged peers, thus steadily increasing the achievement gap throughout the schooling process [94, 96].

### Implications

By indicating that, in normal times, CAI may be more efficient than the well-established method used for Science and Technology (IBL), our findings further legitimize CAI as a way of helping to prevent the disastrous consequences of pandemics on academic learning. However, as also indicated by our data, the benefits of CAI do not occur whatever students’ working memory capacity. The interaction found here between CAI and WM gives us reason to doubt the commercial claims which have multiplied in the absence of solid scientific data since the start of the pandemic, suggesting, for example, that e.learning increases retention rates by 25% to 60%. As our results indicate, even with digital technologies accessible to all (regardless of students’ SES), their educational effectiveness is not necessarily guaranteed, as their benefits for learning may depend on factors such as students’ WM. Failure to take this into account would be to condemn ourselves to an “e.learning illusion” liable to aggravate rather than improve the situation of many students around the globe. In addition to the learning losses characterizing many students during the school holidays (roughly one month of learning on average [99]), students with lower WM capacity would be penalized by inappropriate digital education. There are areas in which this new approach can be implemented successfully, but it is also necessary to be aware that the educational use of CAI and digital technologies in general may have to be nuanced by students’ cognitive characteristics [100].

This research effort, which must be conducted in parallel with the search for Covid-19 vaccines and treatments, is essential if we are to assess the value of CAIs and e.learning in general and not only on the basis of their frequency of use by teachers and students. Likewise, it is essential that the communities concerned (teachers, students and their families, policy makers) discuss their experiences in this area in order not only to try to optimize them but also to identify and/or enrich the most relevant avenues of research and to avoid sterile and possibly also dangerous slogans (as can also be the case with a hastily produced vaccine). Despite the urgency of dealing with the current pandemic, our results therefore suggest that we should not be scared of devoting scientific research to identifying the strengths and weaknesses of the uses of digital technologies and of the currently available services and applications. This is all the more important given that pandemics appear to have been increasing in frequency over the last few decades, and that the adoption of online learning may persist post-pandemic and thus be used more intensively than before. If it has to happen, we stress that policy makers should pay particular attention to the implementation of tutoring techniques in distant learning in addition to the provision of content. In normal times, however, this argument should not be taken as in favor for a “all digitalized” education system, but rather, in favor of a diversification of methods to better address students’ heterogeneity.

### Limitations

Some limitations should be pointed out. First, we were not able to determine exactly which characteristics of CAI specifically tap into WM. Future research is needed to provide clear indications about which features (e.g., navigational constraints, diversity of functionalities) tap into WM and may be further adapted for students with lower WM capacity (in addition to the recommendations provided here on CAI). Second, this study was conducted with middle-school students, meaning that the benefits of CAI might have been underestimated. As shown in previous work, while the effectiveness of IBL is stable across ages [25], CAI effects increase with school grades, with the largest effect sizes being found in postsecondary education [8]. Third, we were limited by the fact that the variety of topics studied here were analysed together as a Science and Technology score. Indeed, although our sample size was large enough to conduct broad–yet robust–analyses, thereby increasing generalizability as in the case of a meta-analysis, it lost in specificity due to the use of smaller and more heterogeneous samples in separate school subjects and experimental conditions, precluding fine-grained–but still robust–analyses. This heterogeneity was directly linked to field constraints and, while we acknowledge that ecological settings do not offer ideal conditions, only this complementary approach can provide a bridge between the laboratory and the real world. A fourth limitation of our study stems for its lack of direct assessment of students’ interest in school subjects and topics. For example, Maltese and Tai (2011) [101] have stressed the importance of students’ early interest in science topics for predicting their enrolment in college science and mathematics courses, with 65% of the students declaring that their interest started before middle school. This is especially important given that IBL is known to enhance students’ motivation to learn and interest in scientific topics [102]. A direct assessment of interest in future studies may be useful in order to gain insights into whether CAI or IBL may differentially benefit students with low interest in science subjects. More generally, a better discrimination of knowledge and skills components in our knowledge tests would have shed light on the differentiated and complementary nature of benefits that CAI and IBL produce on factual knowledge and epistemological thinking [25, 29], which would further legitimate a combined use of these methods. Last but not least, a fifth limitation relates to the lack of a collaborative version of CAI (using CAI in combination with peer-to-peer interactions), which could have clarified our comparison, controlling for the effect of collaboration per se, although the present results do not suggest a particular benefit associated with collaborative work.

## Conclusion

The present study showed the potential of CAI to improve academic performance in Science and Technology compared to IBL, a well-established and popular method of instruction. Compared to IBL, the benefits of CAI were stable across students’ ASC and SES, while being higher for students with higher WM capacity. Despite the overall benefit of CAI, our results suggest that special attention should be paid to the WM demands of CAI, which might require adaptations to the instructional design for students with lower WM capacity.

## Supporting information

S1 File (DOCX)

## References

[1] UNESCO. Education: From disruption to recovery. UNESCO. 2021 Oct. Available from: https://en.unesco.org/covid19/educationresponse

[2] Dreesen T, Akseer S, Brossard M, Dewan P., Giraldo P, Akito K, et al. Promising Practices for Equitable Remote Learning. Emerging lessons from COVID-19 education responses in 127 countries. UNICEF-IRC Innocenti Research Briefs no. 2020–10. 2020 Oct. Available from: https://www.unicef-irc.org/publications/1090-promising-practices-for-equitable-remote-learning-emerging-lessons-from-covid.html.

[3] Watts G. COVID-19 and the digital divide in the UK. Lancet Digit Health. 2020 Aug;2(8):e395–6. doi: 10.1016/S2589-7500(20)30169-2 32835198 PMC7384786

[4] David Y, Sommerlad E. Media and Information in Times of Crisis: The Case of the COVID-19 Infodemic. In: Andrews GJ., Crooks VA., Pearce JR., Messina, JP. (Eds.). COVID-19 and Similar Futures. 2020 Aug. Available from: https://www.springerprofessional.de/media-and-information-in-times-of-crisis-the-case-of-the-covid-1/19275732

[5] Conto MCA, Akseer S, Dreesen T, Kamei A, Mizunoya S, Rigole A. COVID-19: Effects of school closures on foundational skills and promising practices for monitoring and mitigating learning loss. UNICEF-IRC Innocenti Working Papers no. 2020–13. 2020. Available from: https://www.unicef-irc.org/publications/1144-covid19-effects-of-school-closures-on-foundational-skills-and-promising-practices.html

[6] Cotton K. Computer-Assisted Instruction. In: Encyclopedia of Special Education. American Cancer Society; 2008. p. 514–20. Available from: https://onlinelibrary.wiley.com/doi/abs/10.1002/9780470373699.speced0481

[7] Carbonell JR. AI in CAI: An Artificial-Intelligence Approach to Computer-Assisted Instruction. IEEE Trans Man-Mach Syst. 1970 Dec;11(4):190–202.

[8] Kulik JA, Fletcher JD. Effectiveness of Intelligent Tutoring Systems: A Meta-Analytic Review. Rev Educ Res. 2016 Mar 1;86(1):42–78.

[9] Baker RS. Stupid Tutoring Systems, Intelligent Humans. Int J Artif Intell Educ. 2016 Jun 1;26(2):600–14.

[10] OECD. Strengthening online learning when schools are closed: The role of families and teachers in supporting students during the COVID-19 crisis. OECD. 2020 Sep. Available from: https://www.oecd.org/coronavirus/policy-responses/strengthening-online-learning-when-schools-are-closed-the-role-of-families-and-teachers-in-supporting-students-during-the-covid-19-crisis-c4ecba6c/

[11] Timmerman CE, Kruepke KA. Computer-Assisted Instruction, Media Richness, and College Student Performance A previous version of this paper was presented at the 2004 (November) Conference of the National Communication Association, Chicago, IL. Portions of the data presented in this study are also included in Timmerman, C. E., & Kruepke, K. A. (in press). Computer-assisted instruction and college student performance. In B. Gayle, R. Preiss, N. Burrell, & M. Allen (Eds.). Classroom communication and instructional processes: Advances through meta-analysis. Commun Educ. 2006 Jan 1;55(1):73–104.

[12] Campbell DL, Peck DL, Horn CJ, Leigh RK. Comparison of Computer-Assisted Instruction and Print Drill Performance: A Research Note. Educ Commun Technol. 1987 Jan;35(2):95–103.

[13] O’Neil HF Jr., Slawson DA, Baker EL. Design of a domain-independent problem-solving instructional strategy for intelligent computer-assisted instruction. In: Intelligent tutoring systems: Evolutions in design. Hillsdale, NJ, US: Lawrence Erlbaum Associates, Inc; 1991. p. 69–103.

[14] Kulik J. Effects of Using Instructional Technology in Elementary and Secondary Schools: What Controlled Evaluation Studies Say. SRI International (P10446.001). Arlington, VA. 2003 Jan

[15] Trollip S, Ortony A. Real-time simulation in computer-assisted instruction. Instr Sci. 1977 Apr 1;6(2):135–49.

[16] Blake J, Goodman J. Computer-based learning: games as an instructional strategy. ABNF J Off J Assoc Black Nurs Fac High Educ Inc. 1999 Apr;10(2):43–6. 10409946

[17] Finnegan R, Sinatra R. Interactive Computer-Assisted Instruction with Adults. J Read. 1991 Jan 1;35:108–19.

[18] Kulik C-LC, Kulik JA. Effectiveness of computer-based instruction: An updated analysis. Comput Hum Behav. 1991 Jan 1;7(1):75–94.

[19] Ma W, Adesope OO, Nesbit JC, Liu Q. Intelligent tutoring systems and learning outcomes: A meta-analysis. J Educ Psychol. 2014 Nov;106(4):901–18.

[20] Steenbergen-Hu S, Cooper H. A meta-analysis of the effectiveness of intelligent tutoring systems on college students’ academic learning. J Educ Psychol. 2014 May;106(2):331–47.

[21] Pedaste M, Mäeots M,. Siiman LA, de Jong T,. van Riesen SAN, Kamp ET. Manoli CC, et al. Phases of inquiry-based learning: definitions and the inquiry cycle. Educational Research Review, 2015 Sep; 14:47–61.

[22] Bell T, Urhahne D, Schanze S, Ploetzner R. Collaborative Inquiry Learning: Models, Tools, and Challenges. Int J Sci Educ. 2010 Feb 1;32.

[23] Jansen BA. Inquiry Unpacked: An Introduction to Inquiry-Based Learning. Libr Media Connect. 2011 Mar; 29(5): 10–2.

[24] Klahr D, Dunbar K. Dual Space Search During Scientific Reasoning. Cognitive Science. 1988 Jan; 12(1):1–48. Available from: https://onlinelibrary.wiley.com/doi/abs/10.1207/s15516709cog1201_1

[25] Lazonder AW, Harmsen R. Meta-Analysis of Inquiry-Based Learning: Effects of Guidance. Rev Educ Res. 2016 Sep 1;86(3):681–718.

[26] Bittinger ML. A Review Of Discovery. Math Teach. 1968 Feb;61(2):140–6.

[27] Hermann G. Learning by Discovery: A Critical Review of Studies: The Journal of Experimental Education. 1969; 38(1), 58–72. Available from: https://www.tandfonline.com/doi/abs/10.1080/00220973.1969.11011167

[28] OECD. Enquiry-based teaching practices and science performance: Results based on students&apos; reports. OECD average 2015; 2016 Dec. Available from: https://www.oecd-ilibrary.org/education/pisa-2015-results-volume-ii/enquiry-based-teaching-practices-and-science-performance_9789264267510-graph17-en

[29] OECD. PISA 2015 Results (Volume II): Policies and Practices for Successful Schools. OECD (PISA); 2016 Dec. Available from: https://www.oecd-ilibrary.org/education/pisa-2015-results-volume-ii_9789264267510-en

[30] Jerrim J, Oliver M, Sims SG. The relationship between inquiry-based teaching and students’ achievement. New evidence from a longitudinal PISA study in England. Learn Instr. 2019 Jun;61:35–44.

[31] Dobber M, Zwart R, Tanis M, Oers B van. Literature review: The role of the teacher in inquiry-based education. Educ Res Rev. 2017 Nov;22:194–214.

[32] Frey N, Fisher D, Hattie J. Surface, Deep, and Transfer? Considering the Role of Content Literacy Instructional Strategies. J Adolesc Adult Lit. 2017 Jun;60(5):567–75.

[33] Vista A, Kim H, Care E. Use of data from 21^st^ century skills assessments: issues and key principles. Center For Universal Education At Brookings, Unesco IIEP Learning Portal; 2018 Oct. Available from: https://learningportal.iiep.unesco.org/es/biblioteca/use-of-data-from-21st-century-skills-assessments-issues-and-key-principles.

[34] Ventures Brighteye. The European EdTech Funding Report 2020. Brighteye Ventures. 2020 Jan. Available from: https://www.brighteyevc.com/post/european-edtech-funding-report-2020

[35] Graesser A, McNamara D, Graesser A. Self-Regulated Learning in Learning Environments With Pedagogical Agents That Interact in Natural Language. Educ Psychol. 2010 Oct;45(4):234–244.

[36] Killen R. Teaching Strategies for Outcomes-based Education. Juta and Company Ltd; 2007. 412 p.

[37] Kuech R. Collaborative and Interactional Processes in an Inquiry-Based, Informal Learning Environment Journal of Classroom Interaction, 2004, 39(1): 30–41.

[38] Croy MJ. The use of CAI to enhance human interaction in the learning of deductive proof construction. Comput Humanit. 1988 Dec 1;22(4):277–84.

[39] Sosa G, Berger D, Saw A, Mary J. Meta-Analysis Effectiveness of Computer-Assisted Instruction in Statistics: A. Rev Educ Res. 2011 Mar 1;81.

[40] Maor D, Fraser B. An Evaluation of an Inquiry-Based Computer-Assisted Learning Environment. Aust Sci Teach J. 1994;40(4):65–70.

[41] Epstein D, Pinho I da C, Acosta OC, Reategui E. Inquiry-based learning environment using intelligent tutoring system. In: 2013 IEEE Frontiers in Education Conference (FIE). 2013. p. 1072–4.

[42] Sweller J, van Merrienboer JJG, Paas FGWC. Cognitive Architecture and Instructional Design. Educ Psychol Rev. 1998 Sep 1;10(3):251–96.

[43] Cevik V., & Altun A. R. İ. F. Roles of working memory performance and instructional strategy in complex cognitive task performance. Journal of Computer Assisted Learning. 2016; 32: 594–606.

[44] Camos V, Barrouillet P. Working Memory in Development. 2018, Mar. Routledge.

[45] Engle RW. Working Memory and Executive Attention: A Revisit. Perspectives on Psychological Science. 2018 Mar; 13(2): 190–193. doi: 10.1177/1745691617720478 29592654

[46] Engle RW, Tuholski SW, Laughlin JE, Conway ARA. Working memory, short-term memory, and general fluid intelligence: A latent-variable approach. J Exp Psychol Gen. 1999 Sep;128(3):309–31. doi: 10.1037//0096-3445.128.3.309 10513398

[47] Daneman M, Carpenter PA. Individual differences in working memory and reading. J Verbal Learn Verbal Behav. 1980 Aug;19(4):450–66.

[48] Alloway TP, Alloway RG. Investigating the predictive roles of working memory and IQ in academic attainment. J Exp Child Psychol. 2010 May;106(1):20–9. doi: 10.1016/j.jecp.2009.11.003 20018296

[49] Süß H-M, Oberauer K, Wittmann WW, Wilhelm O, Schulze R. Working-memory capacity explains reasoning ability—and a little bit more. Intelligence. 2002 May 1;30(3):261–88.

[50] Unsworth N, Redick T, Heitz R, Broadway J, Engle R. Complex working memory span tasks and higher-order cognition: A latent-variable analysis of the relationship between processing and storage. Mem Hove Engl. 2009 Jul 1;17:635–54. doi: 10.1080/09658210902998047 19536691

[51] Moreno R, Mayer R. Cognitive Principles of Multimedia Learning: The Role of Modality and Contiguity. J Educ Psychol. 1999 Jun 1;91:358–68.

[52] Blom H, Segers E, Knoors H, Hermans D, Verhoeven L. Comprehension and navigation of networked hypertexts. Journal of Computer Assisted Learning. 2018 Jan; 34(3): 306–314

[53] Scharinger C, Kammerer Y, Gerjets P. Pupil Dilation and EEG Alpha Frequency Band Power Reveal Load on Executive Functions for Link-Selection Processes during Text Reading. PLOS ONE. 2015 Jun;10(6):e0130608. doi: 10.1371/journal.pone.0130608 26076026 PMC4468081

[54] Paas FG, Van Merriënboer JJ, Adam JJ. Measurement of Cognitive Load in Instructional Research. Perceptual and motor skills. 1994 Aug; 79(1): 419–430. 10.2466/pms.1994.79.1.419 7808878

[55] Kirschner PA, Sweller J, Clark RE. Why Minimal Guidance During Instruction Does Not Work: An Analysis of the Failure of Constructivist, Discovery, Problem-Based, Experiential, and Inquiry-Based Teaching. Educ Psychol. 2006 Jun;41(2):75–86.

[56] Tanchuk N. Is Inquiry Learning Unjust? Cognitive Load Theory and the Democratic Ends of Education. Journal of Philosophy of Education. 2020 May; 54(5): 1167–1185.

[57] Kaiser I, Mayer J, Malai D. Self-Generation in the Context of Inquiry-Based Learning. Front Psychol. 2018 Dec; 9: 2440 Available from: https://www.frontiersin.org/articles/10.3389/fpsyg.2018.02440/full 30631290 10.3389/fpsyg.2018.02440 PMC6315139

[58] Barrouillet P, Bernardin S, Camos V. Time constraints and resource sharing in adults’ working memory spans. J Exp Psychol Gen. 2004 Mar;133(1):83–100. doi: 10.1037/0096-3445.133.1.83 14979753

[59] Huguet P, Dumas F, Marsh H, Wheeler L, Seaton M, Nezlek J, et al. Clarifying the role of social comparison in the big-fish-little-pond effect (BFLPE): an integrative study. J Pers Soc Psychol. 2009 Jul;97(1):156–70. doi: 10.1037/a0015558 19586246

[60] Marsh HW, Craven RG. Reciprocal Effects of Self-Concept and Performance From a Multidimensional Perspective: Beyond Seductive Pleasure and Unidimensional Perspectives. Perspectives on Psychological Science. 2006 June;1(2):133–163 doi: 10.1111/j.1745-6916.2006.00010.x 26151468

[61] Monteil JM., & Huguet P. Social Context and Cognitive Performance: Towards a Social Psychology of Cognition. Hove, East Sussex: Psychology Press. 1999.

[62] Bandura A. Self-efficacy in changing societies. New York, NY, US: Cambridge University Press; 1995. xv, 334 p.

[63] Bembenutty. The Last Word: An Interview With Herbert W. Marsh: A Leading Voice on Self-Concept, Teaching Effectiveness, and a Force in Quantitative Analysis (Part I)—Journal of Advanced Academics, 2009 Aug;20(4):740–747. Available from: https://journals.sagepub.com/doi/abs/10.1177/1932202X0902000307?journalCode=joac 19610492

[64] Bandura A. Social foundations of thought and action: A social cognitive theory. Englewood Cliffs, NJ, US: Prentice-Hall, Inc; 1986. xiii, 617 p. (Social foundations of thought and action: A social cognitive theory).

[65] Bourdieu P, Passeron JC. Reproduction in education, society and culture, 2^nd^ ed. Thousand Oaks, CA, US: Sage Publications, Inc; 1990. xxvi, 254 p. (Nice R, editor. Reproduction in education, society and culture, 2^nd^ ed).

[66] Moran J, Ferdig RE, Pearson DP, Wardrop J, Blomeyer RL. Technology and Reading Performance in the Middle-School Grades: A Meta-Analysis with Recommendations for Policy and Practice. Journal of Literacy Research, 2008 Mar;40:6–58 Available from: https://journals.sagepub.com/doi/10.1080/10862960802070483

[67] Oswald F. L., McAbee S. T., Redick T. S., & Hambrick D. Z. The development of a short domain-general measure of working memory capacity. Behavior Research Methods. 2015 Dec;47: 1343‑1355. doi: 10.3758/s13428-014-0543-2 25479734

[68] Marsh HW. The structure of academic self-concept: The Marsh/Shavelson model. J Educ Psychol. 1990;82(4):623–36.

[69] Institut national de la statistique et des études économiques. Nomenclatures des professions et catégories socioprofessionnelles des emplois salariés des employeurs privés et publics. INSEE 2018; Available from https://www.insee.fr/fr/information/2497958.

[70] Bray J, Schlenger W, Zarkin G, Galvin D. Analyzing Data from Nonrandomized Group Studies. RTI Press publication No. MR-0008-0811. Research Triangle Park, NC: RTI International. 2008 Nov. Available from 10.3768/rtipress.2008.mr.0008.0811.

[71] Castro-Schilo L., & Grimm K. J. Using residualized change versus difference scores for longitudinal research. Journal of Social and Personal Relationships. 2017 Dec; 35: 32–58.

[72] Xiao Z., Higgins S., & Kasim A. An Empirical Unravelling of Lord’s Paradox. The Journal of Experimental Education. 2017 Sep; 87: 17–32.

[73] R Core Team. R: A Language and Environment for Statistical Computing. R Foundation for Statistical Computing, Vienna. 2013; Available from http://www.R-project.org/

[74] Fox J, & Weisberg S. An R Companion to Applied Regression, Third edition. Sage, Thousand Oaks CA. 2019.

[75] Bates D, Mächler M, Bolker B, Walker S. Fitting Linear Mixed-Effects Models Using lme4. J Stat Softw. 2015 Oct;67(1):1–48.

[76] Ministères de l’Education Nationale, de l’Enseignement Supérieur, de la Recherche et de l’Innovation: Direction de l’évaluation, de la prospective et de la performance. Repère et références statistiques Enseignement, Formation, Recherche. 2018 Aug; Available from https://cache.media.enseignementsup-recherche.gouv.fr/file/RERS_2018/83/2/depp-2018-RERS-web_986832.pdf

[77] National Centre for Educational Statistics. Digest of Education Statistics: 2018. 2018; Available from https://nces.ed.gov/programs/digest/d13/tables/dt13_203.10.asp.

[78] Botsas G, Grouios G. Computer Assisted Instruction of Students with ADHD and Academic Performance: A Brief Review of Studies Conducted Between 1993 and 2016, and Comments. European Journal of Special Education Research. 2017 Jan;2(6).

[79] Osman G, Duffy TM, Chang J, Lee J. Learning through Collaboration: Student Perspectives. Asia Pac Educ Rev. 2011 Dec;12(4):547–58.

[80] Chang Y, Brickman P. When Group Work Doesn’t Work: Insights from Students. CBE Life Sci Educ. 2018 Sep;17(3):ar52. doi: 10.1187/cbe.17-09-0199 30183565 PMC6234829

[81] Godwin KE, Seltman H, Almeda MVQ, Kai S, Baker RS, Fisher AV. The Variable Relationship Between On-Task Behavior and Learning. Cog Sci. 2016. 29359204

[82] Van der Kleij FM, Feskens RCW, Eggen TJHM. Effects of Feedback in a Computer-Based Learning Environment on Students’ Learning Outcomes: A Meta-Analysis. Rev Educ Research. 2015 Dec; 85(4): 475–511. Available from: https://www.researchgate.net/publication/272923307_Effects_of_Feedback_in_a_Computer-Based_Learning_Environment_on_Students’_Learning_Outcomes_A_Meta-Analysis

[83] Hachfeld A, Lazarides R. The relation between teacher self-reported individualization and student-perceived teaching quality in linguistically heterogeneous classes: an exploratory study. Eur J Psychol Educ. 2020 Oct. Available from: https://kops.uni-konstanz.de/handle/123456789/51627

[84] Hovardas T, Tsivitanidou OE, Zacharia ZC. Peer versus expert feedback: An investigation of the quality of peer feedback among secondary school students. Comput Educ. 2014 Feb;71:133–52.

[85] Perrin D. Facilitating Student Learning Through Contextualization: A Review of Evidence. Community College Review. 2011 Jul; 39(3): 268–295

[86] Font JTP, Argüello M. Stories or Scenarios: Implementing Narratives in Gamified Language Teaching. In: GamiLearn. 2019;2427.

[87] Madan CR, Singhal A. Using actions to enhance memory: effects of enactment, gestures, and exercise on human memory. Front Psychol. 2012 Nov 19;3:507. doi: 10.3389/fpsyg.2012.00507 23293612 PMC3536268

[88] Steffens MC, von Stülpnagel R, Schult JC. Memory Recall After “Learning by Doing” and “Learning by Viewing”: Boundary Conditions of an Enactment Benefit. Front Psychol. 2015 Dec;6:1907. doi: 10.3389/fpsyg.2015.01907 26733905 PMC4681778

[89] Rodrigues C, Rodrigues J, Ferro M and Biagi F. Digital technologies and learning outcomes of students from low socio-economic background: An Analysis of PISA 2015. EUR 28688 EN. Luxembourg (Luxembourg): Publications Office of the European Union; 2017. JRC106999

[90] Zhan Z., & Mei H. Academic self-concept and social presence in face-to-face and online learning: Perceptions and effects on students’ learning achievement and satisfaction across environments. Computers & Education, 2013 Nov; 69: 131–138.

[91] de Jong T. Cognitive load theory, educational research, and instructional design: Some food for thought. Instr Sci. 2009 Aug;38(2):105–34.

[92] Wegner DM. Transactive Memory: A Contemporary Analysis of the Group Mind. 1987. In Theories of group behavior. Springer, New York, NY p. 185–208.

[93] Hollingshead AB, Gupta N, Yoon K, Brandon DP. Transactive memory theory and teams: Past, present, and future. In: Theories of team cognition: Cross-disciplinary perspectives. New York, NY, US: Routledge/Taylor & Francis Group; 2012. p. 421–55.

[94] Rulke DL, Rau D. Investigating the Encoding Process of Transactive Memory Development in Group Training. Group & Organization Management. 2000 Dec; 25(4): 373–396.

[95] Lewis K, Herndon B. Transactive memory systems: Current issues and future research directions. Organ Sci. 2011 Sep; 22(5): 1254–65.

[96] Gray SA, Chaban P, Martinussen R, Goldberg R, Gotlieb H, Kronitz R, et al. Effects of a computerized working memory training program on working memory, attention, and academics in adolescents with severe LD and comorbid ADHD: a randomized controlled trial. J Child Psychol Psychiatry. 2012 Dec;53(12):1277–84. doi: 10.1111/j.1469-7610.2012.02592.x 22978357

[97] Paas F, van Merriënboer JJ. Cognitive-Load Theory: Methods to Manage Working Memory Load in the Learning of Complex Tasks. Current Directions in Psychological Science, 2020 Jul; 29(4): 394–398.

[98] Mingo I, Bracciale R. The Matthew Effect in the Italian Digital Context: The Progressive Marginalisation of the “Poor.” Soc Indic Res. 2018 Jan 1;135(2):629–59.

[99] Cooper H, Nye B, Charlton K, Lindsay J, Greathouse S. The Effects of Summer Vacation on Achievement Test Scores: A Narrative and Meta-Analytic Review. Review of educational research, 1996 Sep; 66(3): 227–268.

[100] Leroux G, Monteil JM, Huguet P. Technology for learning: A critical review of the meta-analysis literature. L’Année Psychologique, 2017 117(4): 433–465. Available from: 10.4074/S0003503317000586.

[101] Maltese AV, Tai R. Pipeline persistence: Examining the association of educational experiences with earned degrees in STEM among U.S. students. Science Education. 2011 May;95(5):877–907. Available from: https://onlinelibrary.wiley.com/doi/abs/10.1002/sce.20441

[102] Wang PH, Wu PL, Yu KW, Lin YX. Influence of Implementing Inquiry-based Instruction on Science Learning Motivation and Interest: A Perspective of Comparison—Procedia—Social and Behavioral Sciences. 2015 Feb; 174(12):1292–1299 Available from: https://www.sciencedirect.com/science/article/pii/S1877042815008022

