# Supplementary material for: Computer-assisted instruction versus inquiry-based learning: The importance of working memory capacity
Source: PLoS One. 2021 Nov 9;16(11):e0259664. doi: 10.1371/journal.pone.0259664 (PMC8577743; doi:10.1371/journal.pone.0259664)
Supplement: S1 File — (DOCX) [file pone.0259664.s001.docx]

**Supporting Information**

For

**“Comparing traditional Computer-Assisted Instruction and Inquiry-Based Learning methods in a French cohort of middle schoolers: the importance of working memory capacity”**

Chevalère J., Cazenave, L., Berthon, M., Martinez, R., Mazenod, V., Borion, M.C., Pailler, D., Rocher, N., Cadet, R., C. Lenne, C., Maïonchi-Pino, N., & Huguet, P.

Includes: S1 Appendix, S2 Appendix, S3 Appendix, S4 Appendix

**S1 Appendix. Example of a problem-solving exercise (a.) and feedback (b.) within a narrative scenario.** The narrative scenario is entitled “Banana Jones and the crystal statuette” and is used in middle school Physics-Chemistry CAI.

a.


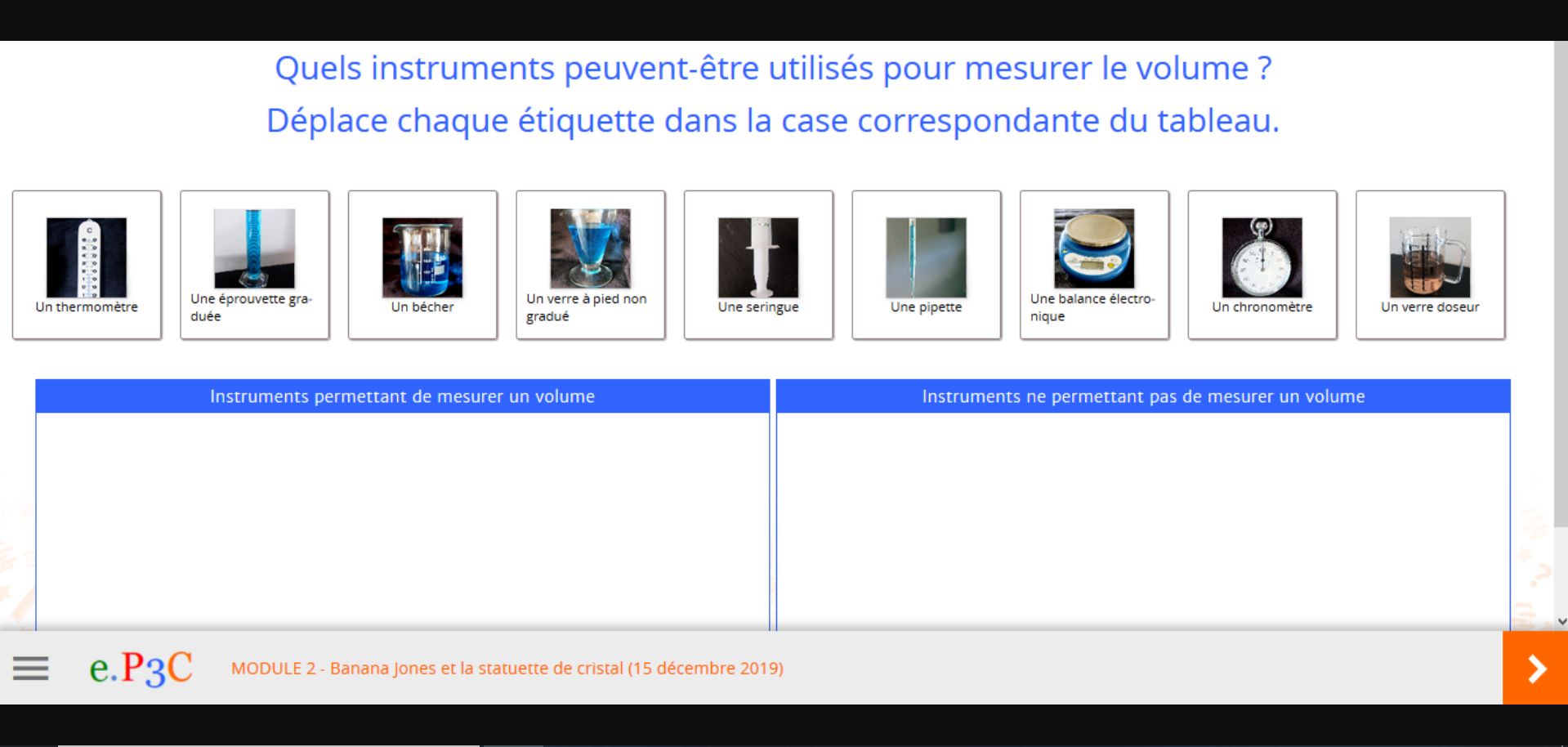


b.


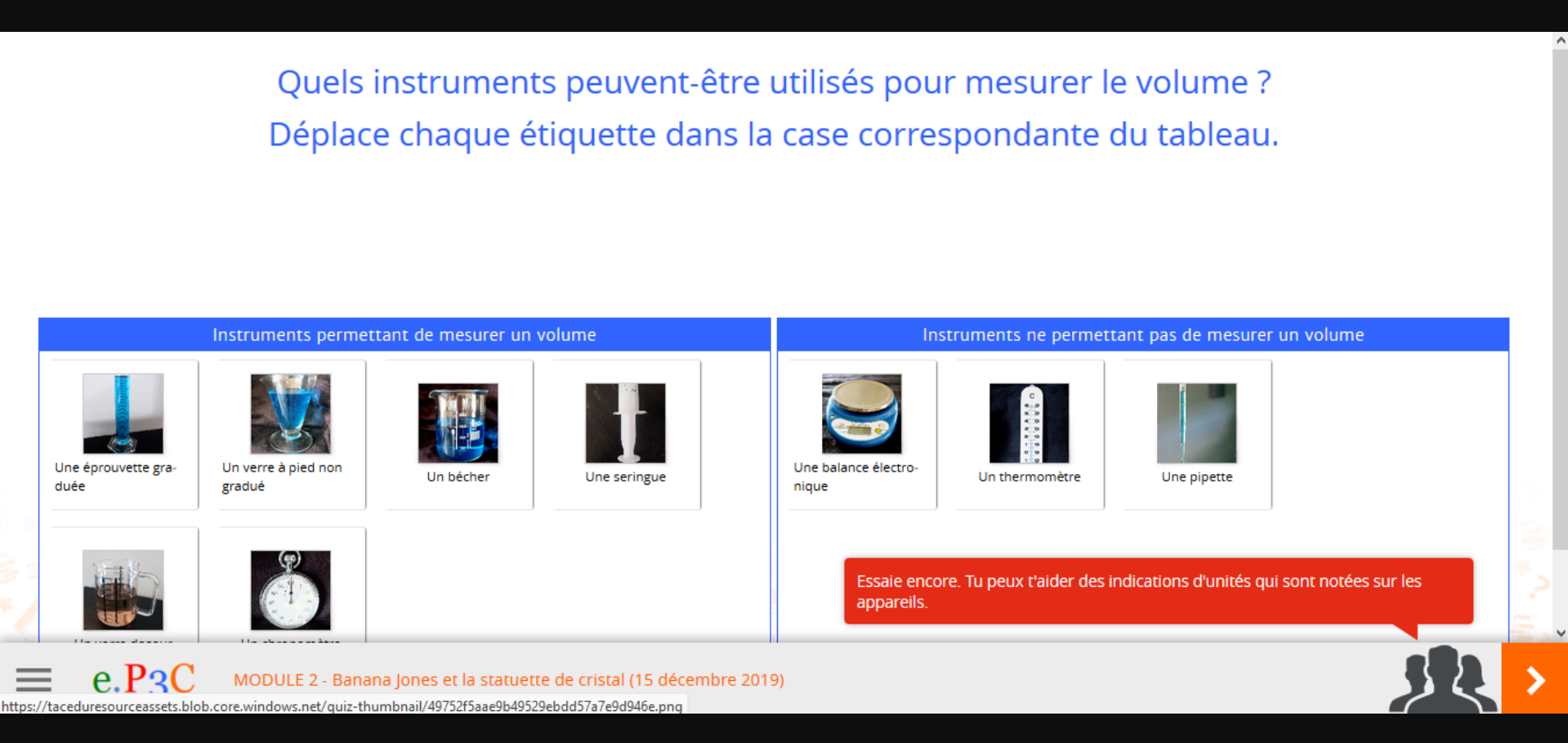


*Note*. Students are instructed to sort the instruments by placing them in the correct category. In panel b. the student receives a negative feedback and a hint. The narrative scenario invites students to solve a series of problems that would help Banana Jones solve the mystery of the crystal statuette.

**S2 Appendix. Example of a scaffolding approach (a. to h.) with a virtual tutor middle school Physics-Chemistry CAI.** The approach guides student towards understanding the notion of volume in relation to a measuring cylinder. The step-by-step problem-solving is contextualized within the “Banana Jones and the crystal statuette” narrative scenario.

a.


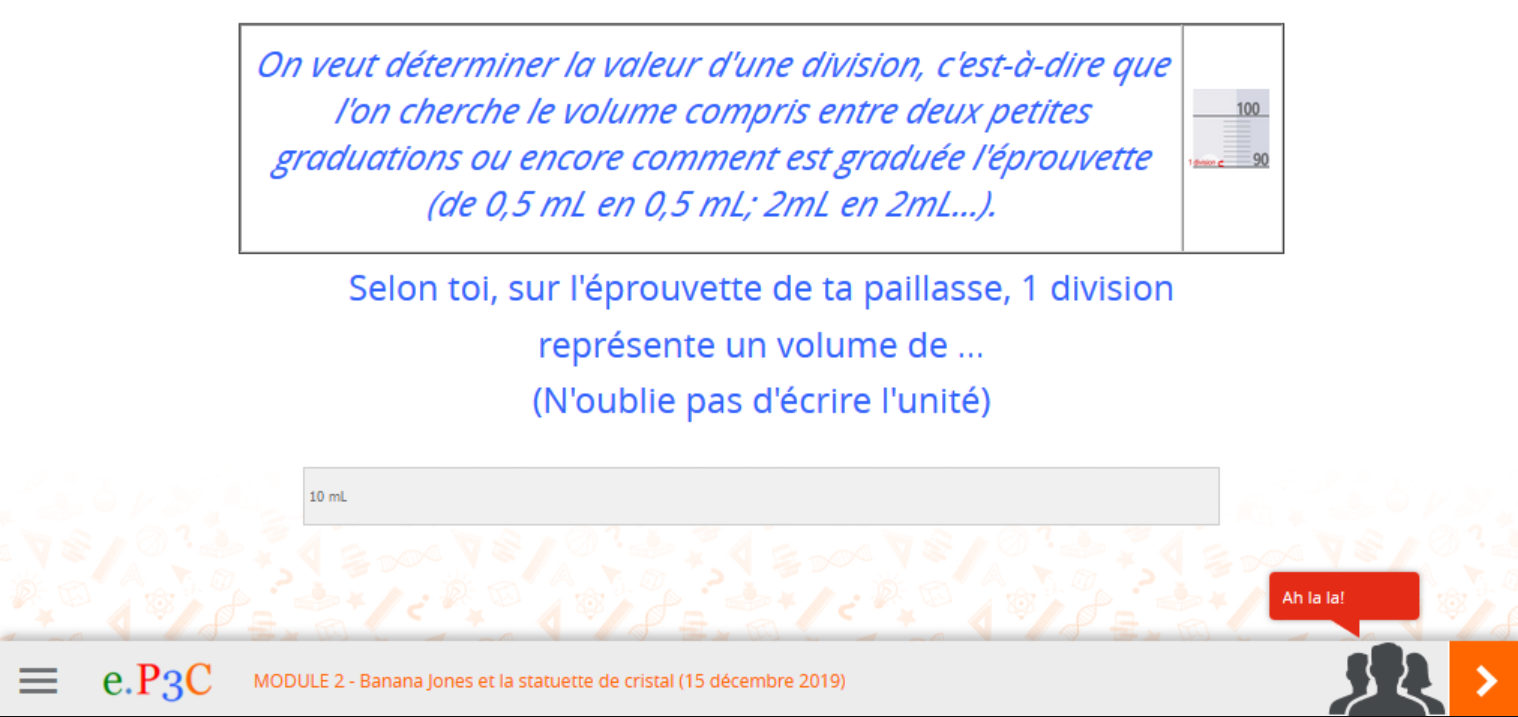


*Note.* The student receives a negative feedback

b.


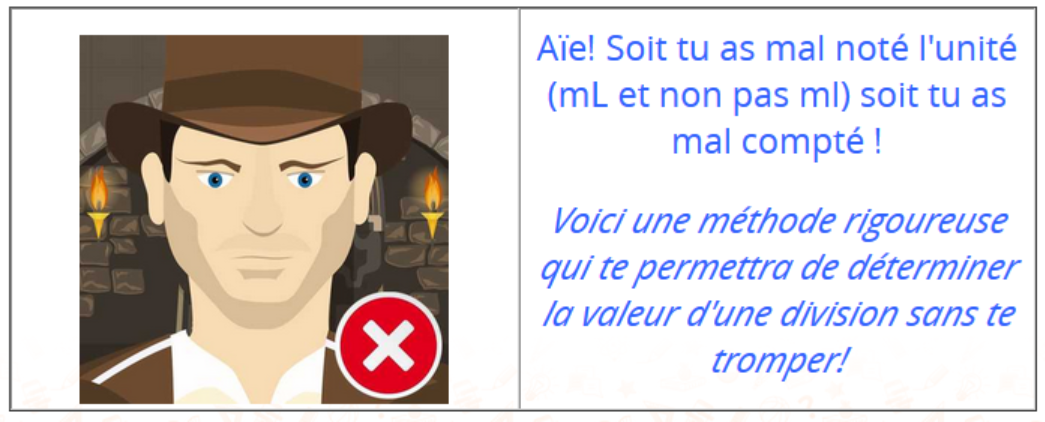


*Note*. The virtual tutor helps the student by proposing a method to solve the problem.

c.


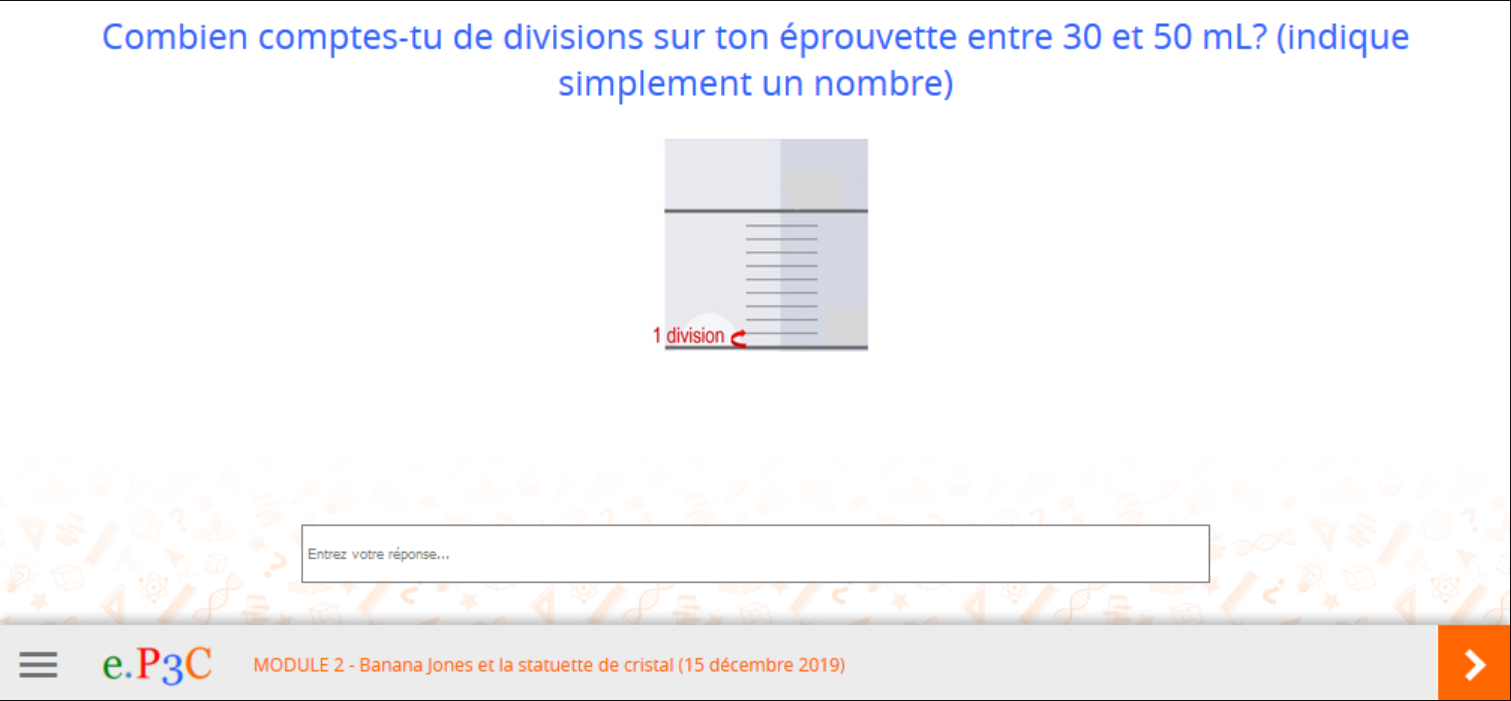


*Note*. The method is proposed.

d.


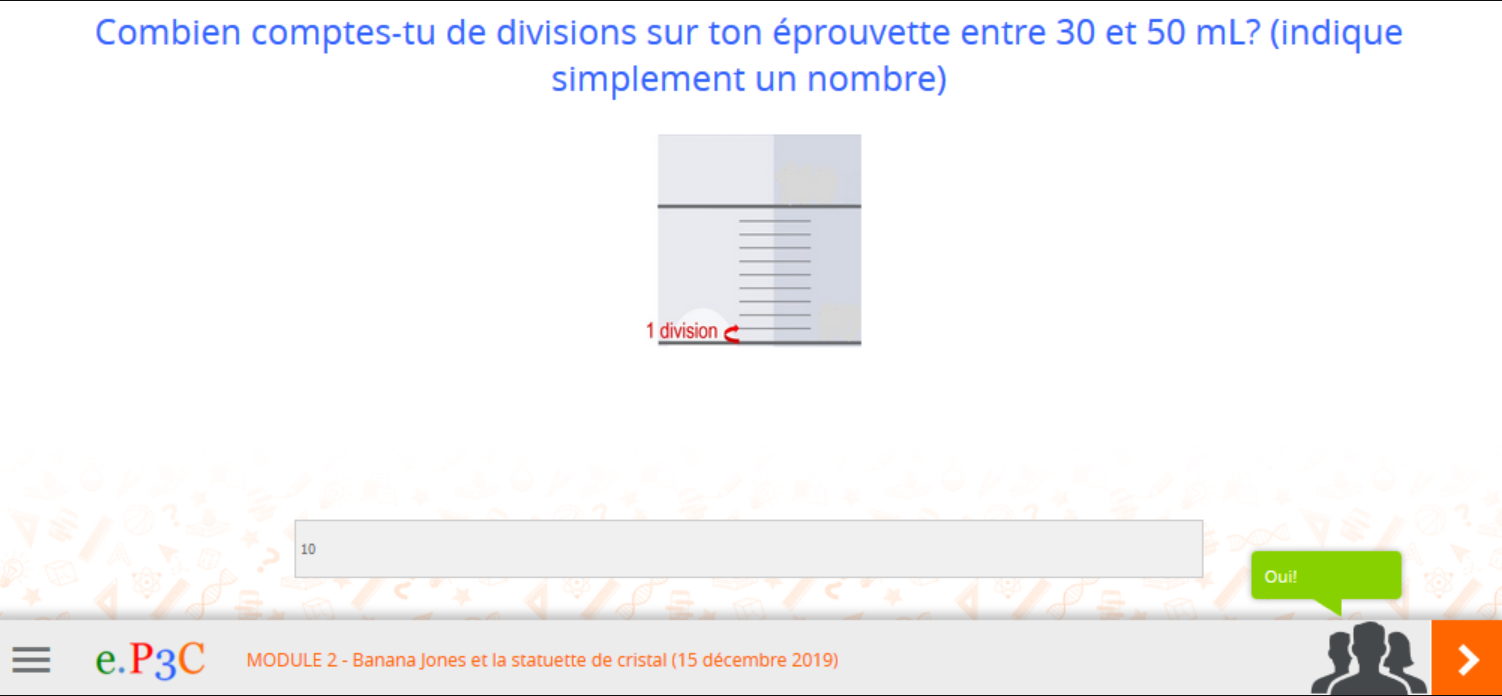


*Note*. The student solves the first step and received a positive feedback.

e.


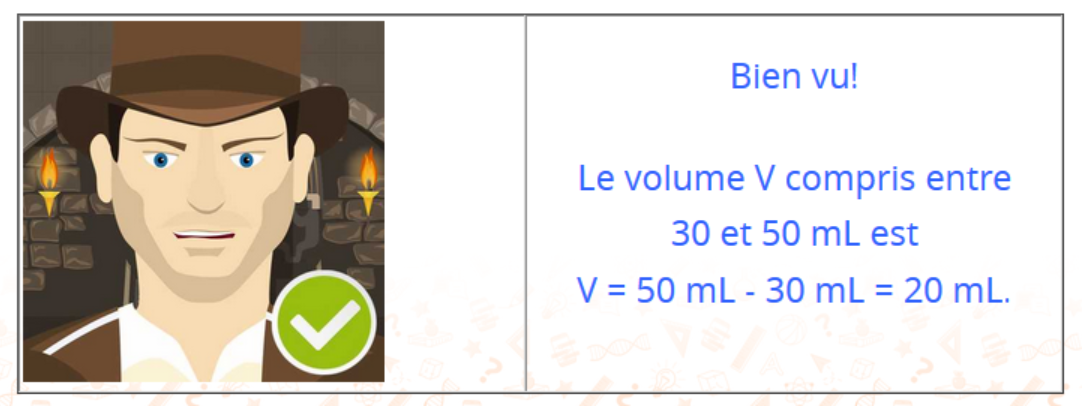


*Note*. The virtual tutor gives information about the next step.

f.


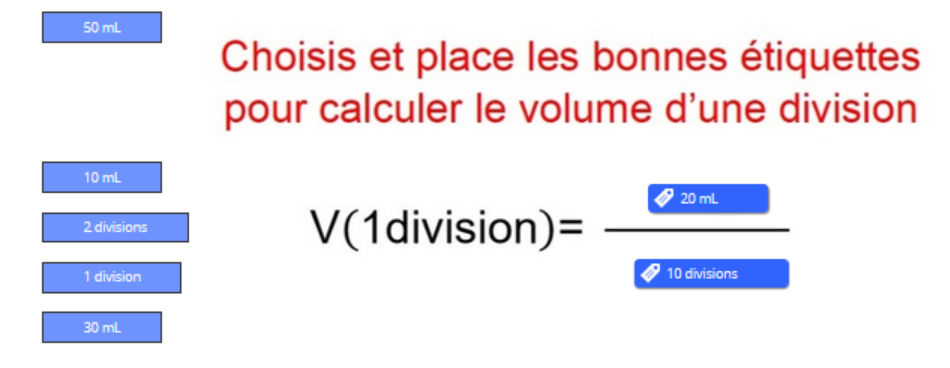


*Note*. The proposed method continues with the second step.

g.


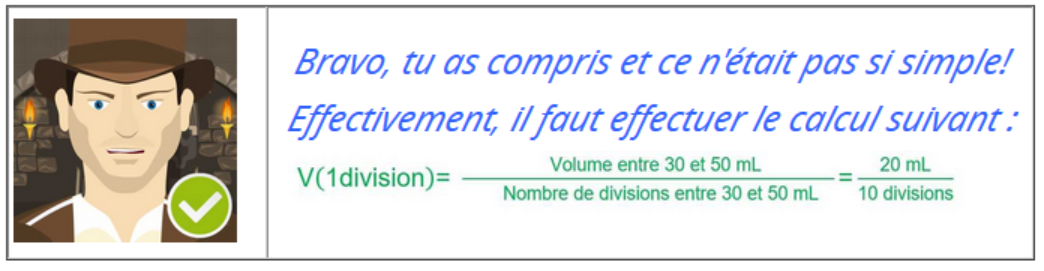


*Note*. The student receives a positive feedback confirming her correct answer with a short explanation.

h.


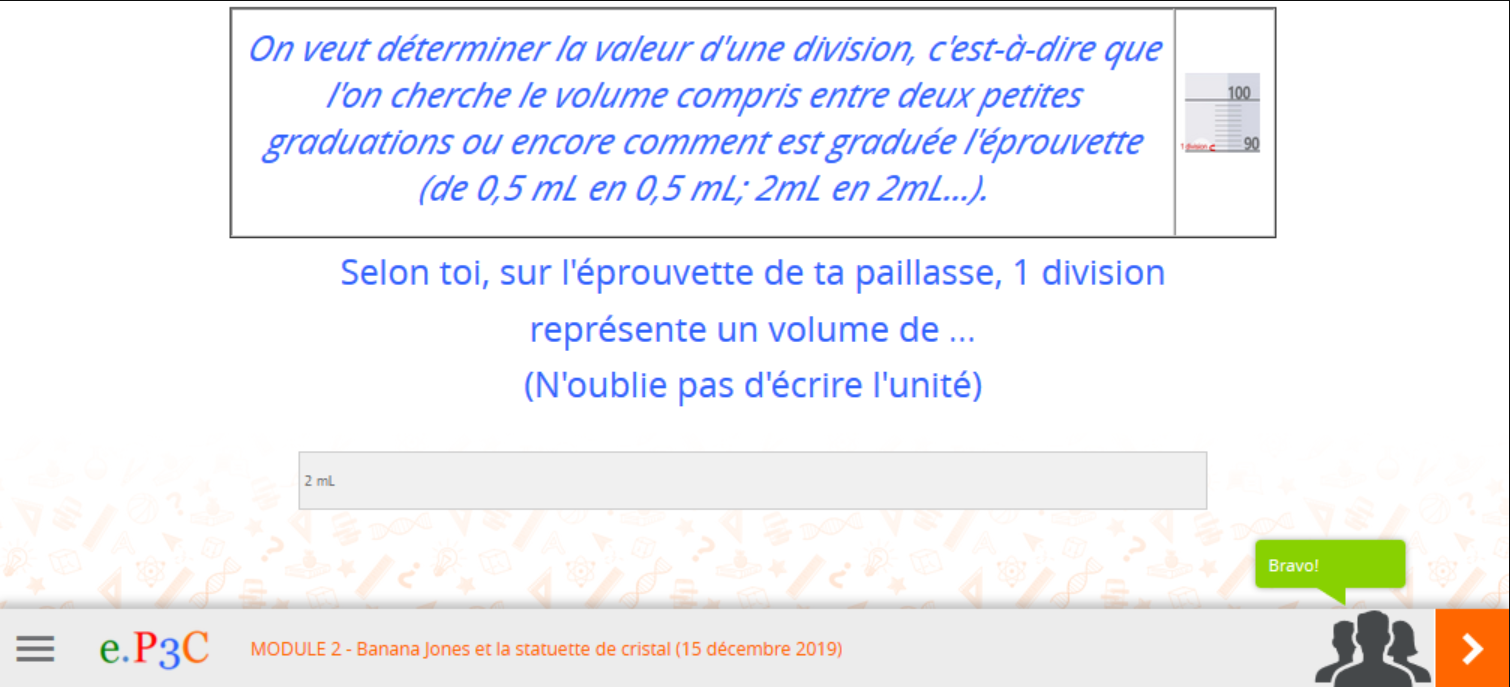


*Note*. The student is presented again with the first question, she answers correctly and receives a positive feedback.

**S3 Appendix. The knowledge post-test in middle Physics-Chemistry on the topic “mass and volume”.** In French language.

**Evaluation sommative de Physique-Chimie, groupe collège**

**Distinction entre masse et volume**

**Objectifs de compétences et de connaissances visés au cours de la séquence :**

- Savoir définir la masse et le volume.
- Savoir mesurer une masse et un volume d’un liquide et d’un solide.
- Connaitre le vocabulaire des appareils de mesure, les unités.
- Savoir convertir sur des unités courantes de masse et de volume.
- Savoir utiliser les connaissances et compétences de masse et volume dans une situation complexe (communication, expérimentale, élaboration et mise en place d’un protocole, …) : densité, proportionnalité entre masse et volume pour une substance donnée (masse volumique).

1. **On considère les trois situations suivantes :**

**a**

1 g

**b**

20 g

Situation **a** Situation **b**

**c**

100 g

Situation **c**

- 1. Quel est l'objet le plus lourd ?

 a  b  c

- 1. Q uel est l'objet le plus gros ?

 a  b c

- 1. Q uel est l'objet ayant la masse la plus importante ?

 a  b  c

- 1. Q uel est l'objet ayant le plus grand volume ?

 a  b  c

1. **On verse un liquide vert dans une éprouvette (A) et une pipette (B).**

**A B**


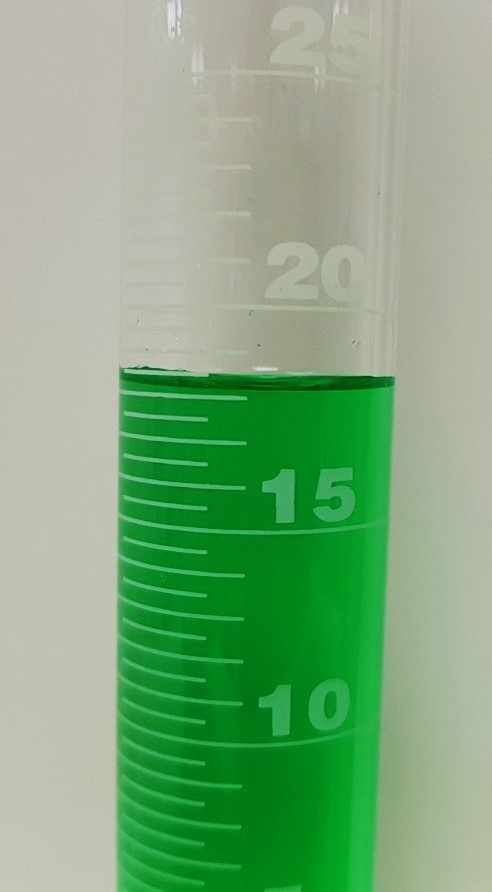

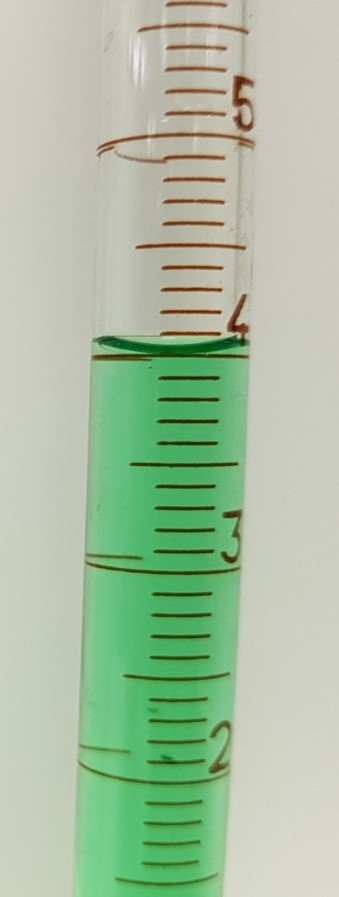


**Eprouvette graduée (unité utilisée : mL)**

**Pipette graduée (unité utilisée : mL)**

- 1. Q ue mesure l’élève en utilisant ces récipients ?

 La masse de liquide  le volume de liquide  la température du liquide

- 1. Q uel est le nom donné par les scientifiques à la forme que prend la surface libre

du liquide ?

 le ménisque  la courbe  le creux  la vague

- 1. Q uelle est la valeur de la mesure pour l’éprouvette A ?

 18,5 mL  21 g  19 mL  18 mL  21 mL

- 1. Q uelle est la valeur de la mesure pour la pipette B ?

 4,1 mL  34 g  4 mL  4,2 mL  4,1 g

1. **Un élève réalise deux manipulations sur un échantillon de métal représenté par le cube noir.**

**Voici les schémas de ses manipulations :**

| **Manipulation 1** | **Manipulation 2** |
| --- | --- |
| **37,8 g** | **Liquide** |

- 1. L’élève réalise la manipulation 1 pour mesurer :


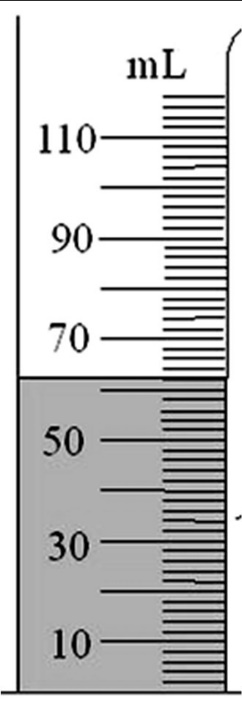

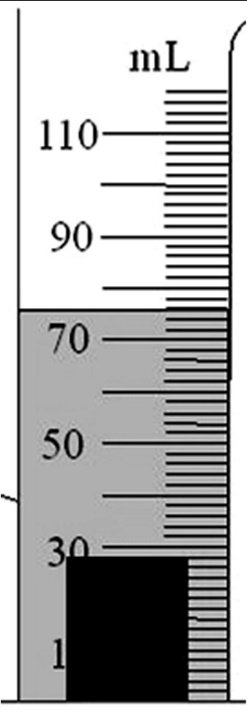


 la masse de l’échantillon de métal.

 le volume de l’échantillon de métal.

 la température de l’échantillon de métal.

- 1. L’élève réalise la manipulation 2 pour mesurer :

 la masse de l’échantillon de métal.

 le volume de l’échantillon de métal.

 la température de l’échantillon de métal.

- 1. Quelle est la valeur du volume de l’échantillon de métal ?

 37,8 g  62 mL  28 mL  76 mL  14 mL

1. **Un élève souhaite mesurer la masse de lait contenu dans un verre.**
   1. Quel appareil doit-il utiliser ?

 une balance  une éprouvette  un thermomètre.

- 1. L’élève réalise différentes étapes.

A toi de les mettre dans l’ordre en mettant « 1 » pour la 1ère étape puis « 2 » puis etc.

| **Etape** | **Ordre de l’étape** |
| --- | --- |
| L’élève lit l’indication sur l’écran de l’appareil. |  |
| L’élève allume l’appareil. |  |
| L’élève appuie sur le bouton TARE de l’appareil. |  |
| L’élève vide le lait dans le récipient. |  |
| L’élève place un bécher vide sur l’appareil. |  |

- 1. L’élève lit sur l’appareil un nombre égal à 85.

D’après vous en quelle unité est exprimée cette mesure. Attention à bien rester

cohérent avec la quantité de matière possible que peut contenir un verre.

 85 kg  85 g  85 mL  85 L

1. **Complète le tableau : pour chaque matériau, coche la bonne proposition flotte sur l’eau ou coule dans l’eau.**

| **Matériau** | **Densité** | **Flotte sur l’eau** | **Coule dans l’eau** |
| --- | --- | --- | --- |
| **Fer**  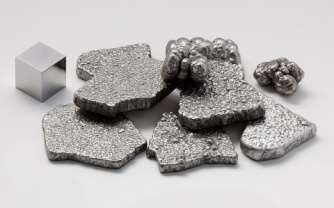 | **7,87** |  |  |
| **Liège**  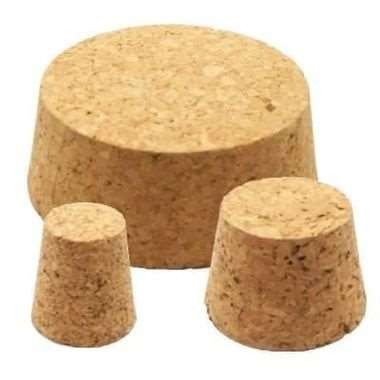 | **0,26** |  |  |
| **Sapin**  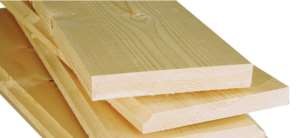 | **0,42** |  |  |
| **Verre**  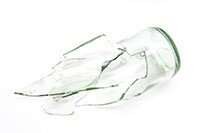 | **2,5** |  |  |

1. **Lydie, une élève, a devant elle une bouteille contenant un liquide incolore non identifié. Elle souhaite savoir si ce liquide est de l’eau ou pas.**

**Malheureusement, elle ne possède pas de sulfate de cuivre anhydre pour tester ce liquide. Elle décide donc de déterminer la masse d’un litre de ce liquide.**

- 1. Q uelle est la masse d’un litre d’eau dans les conditions usuelles de notre

environnement ?

 10 kg  1 g  1 kg  1000 mL

- 1. Dans le laboratoire, Lydie a à sa disposition une balance électronique, une éprouvette graduée de 100 mL et la bouteille contenant le liquide inconnu.

Elle pèse la masse de 50 mL de ce liquide et trouve 42 g.

Quelle est la masse d’un litre de ce liquide inconnu ?

 840 kg  0,84 kg  420 kg  0,42 g

- 1. E n t’aidant du tableau ci-dessous, identifie ce liquide. Il s’agit de :

 alcool méthylique  essence  éthanol  huile  sapin  glycérol

|  |  |
| --- | --- |
| **Substances à 20 °C** | **Masse d’1L de substance (en kg)** |
|  |  |
| **sapin** | **0,42** |
| **alcool méthylique** | **0,79** |
| **essence** | **0,84** |
| **éthanol (alcool)** | **0,87** |
| **huile** | **0,92** |
| **glycérol** | **1,26** |

**S4 Appendix. The official content of the French National Educational Programme for each discipline in middle school** (Physics-Chemistry, Earth and Life Sciences, and Technology).

**Physics-Chemistry**

[**https://eduscol.education.fr/296/physique-chimie-cycle-4**](https://eduscol.education.fr/296/physique-chimie-cycle-4)

**Earth and Life Sciences**

[**https://eduscol.education.fr/293/sciences-de-la-vie-et-de-la-terre-cycle-4**](https://eduscol.education.fr/293/sciences-de-la-vie-et-de-la-terre-cycle-4)

**Technology**

[**https://eduscol.education.fr/282/technologie-cycle-4**](https://eduscol.education.fr/282/technologie-cycle-4)
